# Interleukin-4 activated macrophages mediate immunity to filarial helminth infection by sustaining CCR3-dependent eosinophilia

**DOI:** 10.1371/journal.ppat.1006949

**Published:** 2018-03-16

**Authors:** Joseph D. Turner, Nicolas Pionnier, Julio Furlong-Silva, Hanna Sjoberg, Stephen Cross, Alice Halliday, Ana F. Guimaraes, Darren A. N. Cook, Andrew Steven, Nico Van Rooijen, Judith E. Allen, Stephen J. Jenkins, Mark J. Taylor

**Affiliations:** 1 Research Centre for Drugs & Diagnostics, Liverpool School of Tropical Medicine, Liverpool, United Kingdom; 2 VU University Medical Center, Department of Molecular Cell Biology and Immunology, Amsterdam, Netherlands; 3 Faculty of Biology, Medicine & Health, University of Manchester, Manchester, United Kingdom; 4 MRC Centre for Inflammation Research, University of Edinburgh, Edinburgh, United Kingdom; University of Medicine & Dentistry New Jersey, UNITED STATES

## Abstract

Eosinophils are effectors in immunity to tissue helminths but also induce allergic immunopathology. Mechanisms of eosinophilia in non-mucosal tissues during infection remain unresolved. Here we identify a pivotal function of tissue macrophages (Mϕ) in eosinophil anti-helminth immunity using a BALB/c mouse intra-peritoneal *Brugia malayi* filarial infection model. Eosinophilia, via C-C motif chemokine receptor (CCR)3, was necessary for immunity as CCR3 and eosinophil impairments rendered mice susceptible to chronic filarial infection. Post-infection, peritoneal Mϕ populations proliferated and became alternatively-activated (AAMϕ). Filarial AAMϕ development required adaptive immunity and interleukin-4 receptor-alpha. Depletion of Mϕ prior to infection suppressed eosinophilia and facilitated worm survival. Add back of filarial AAMϕ in Mϕ-depleted mice recapitulated a vigorous eosinophilia. Transfer of filarial AAMϕ into Severe-Combined Immune Deficient mice mediated immunological resistance in an eosinophil-dependent manner. Exogenous IL-4 delivery recapitulated tissue AAMϕ expansions, sustained eosinophilia and mediated immunological resistance in Mϕ-intact SCID mice. Co-culturing *Brugia* with filarial AAMϕ and/or filarial-recruited eosinophils confirmed eosinophils as the larvicidal cell type. Our data demonstrates that IL-4/IL-4Rα activated AAMϕ orchestrate eosinophil immunity to filarial tissue helminth infection.

## Author summary

Helminths parasitize approximately one quarter of the global population. Medically-important helminths, including filariae responsible for elephantiasis and river blindness, are targeted for elimination as a public health problem. Currently there are no vaccines or immunotherapeutics available for filarial worms or other human helminth pathogens. Here we define a cellular mechanism whereby the interlukin-4 dependent activation of tissue macrophages are essential to sustain the recruitment of larvicidal eosinophil granulocytes, leading to immunity against filarial infection at a sterile tissue site of parasitism. This work delineates the relative non-redundant functional roles of both myeloid cell types in ‘type-2’ immunity to helminth infection. The study represents a mechanistic advance in our understanding of how immunity operates against metazoan macroparasites invading sterile tissues and may be used in the rational design of new therapeutics to limit helminth disease.

## Introduction

Infections by helminth parasites are frequently accompanied by overt eosinophilia at parasitized tissue niches[1]. In animal models of infection, eosinophils are functionally important in the immune effector response directed at tissue-invading helminths[2–8] but can also drive pathology[2] and are implicated in immune regulation potentially via the provision of T-cell polarizing signals[9, 10]. Antibody-dependent cellular cytotoxicity (ADCC) and granule-released products have been implicated as the mechanism by which eosinophils mediate parasite helminth larval attrition both *in vitro*[11, 12] and *in vivo*[4, 7, 8]. Corroborating eosinophilic immunity demonstrable in rodent models, clinical studies have identified that interleukin-5, a growth factor supporting eosinophilia, is a correlate of resistance to helminth re-infection[13, 14]. Also, tissue IL-5 and eosinophilia at the site of larval establishment have been demonstrated in experimental human challenge models[15, 16]. Whilst the importance of eosinophils in immunity to tissue-invading helminth parasites is well-defined, much less is understood about the cellular mechanism by which a tissue eosinophilia in parasitized tissues is coordinated and maintained.

Macrophages (Mϕ), polarised to non-classical ‘alternatively activated’ (AAMϕ) phenotypes, are an additional cellular hallmark of helminth infection[17]. However, unlike the immune-effector activity of eosinophils, AAMϕ differentiated from recruited blood monocytes have been identified as mediators of host-protective, wound-healing T helper 2 (Th2) responses to rapidly repair lesions caused by helminth larvae as they migrate through barrier sites (the skin, lungs and gut)[18–21]. An associated AAMϕ function of promoting immunoregulation, including during chronic helminth infection, has been demonstrated[9, 20, 22–25]. Thus, a paradigm of AAMϕ function is to regulate Th2 inflammation and initiate wound healing during parasitological assault.

AAMϕ are also generated at non-barrier, ‘sterile’ sites of infection by tissue helminths, such as filarial nematodes, where they proliferate from resident Mϕ in response to interleukin (IL) 4 / IL-13 signals[26, 27]. Therefore, at sterile sites of infection, tissue-proliferating AAMϕ may have distinct immune functions other than wound healing and immunoregulation, during an initial response to helminth infection.

In this investigation, we delineate the functions of eosinophils and local AAMϕ populations in immunity against *Brugia malayi* larvae in a murine, Th2-adaptive immune peritoneal infection model. We determine that IL-4-dependent alternative activation and expansion of Mϕ are essential to regulate eosinophil-dependent immunity to filarial helminth infection via amplifying and sustaining CCR3-dependent tissue eosinophilia.

## Results

### CCR3-dependent tissue eosinophilia is necessary for immunity to *B*. *malayi* invading larvae

**Fig 1:**
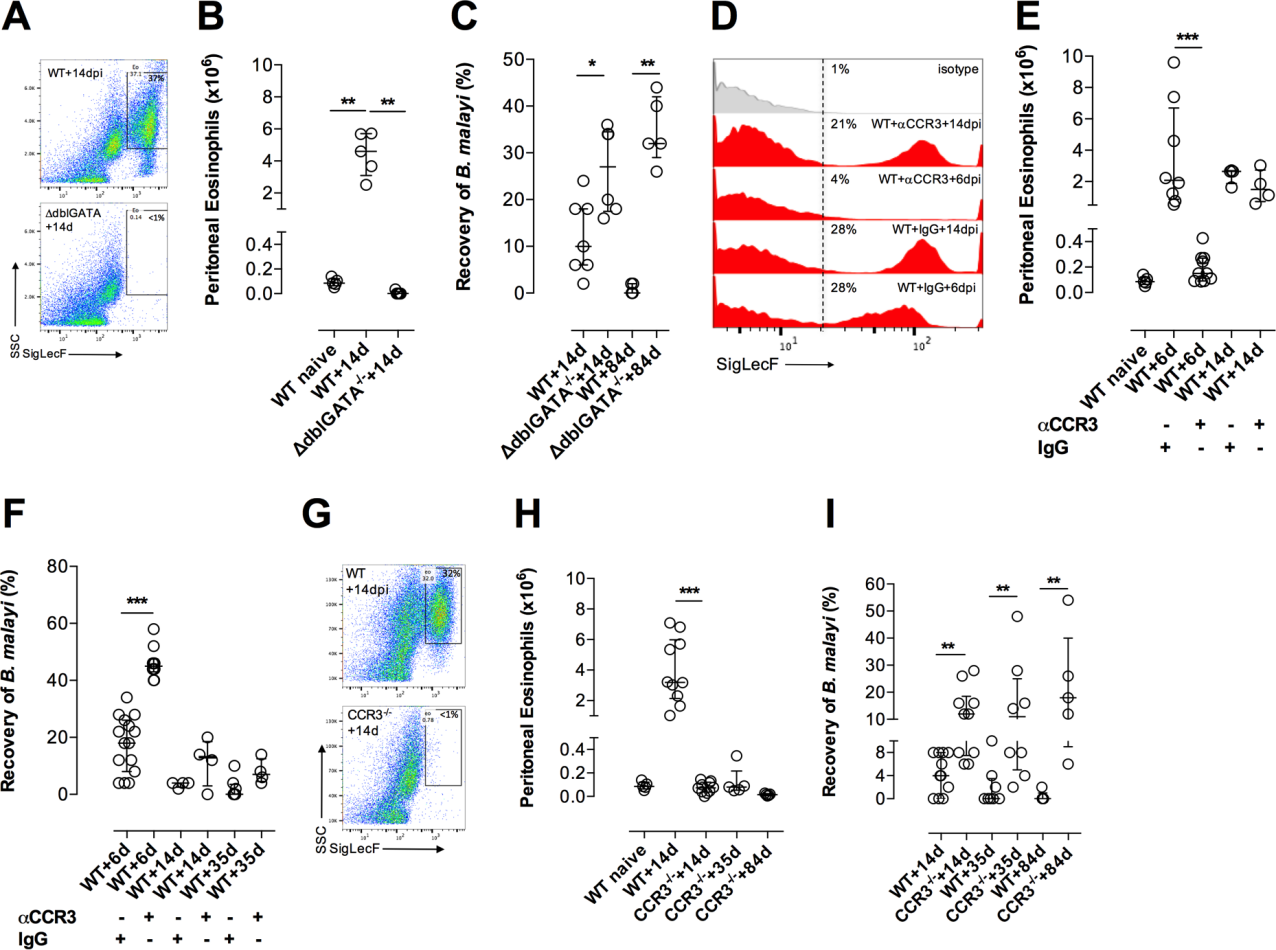
CCR3-dependent tissue eosinophilia is required for immunity to *B*. *malayi*. Enumeration of peritoneal eosinophils **(A,B,D,E,G,H)** and % recoveries of motile *B*. *malayi* in BALB/c WT compared with ΔdblGATA deficient mice **(C)**, in BALB/c WT mice treated with intraperitoneal (ip) rat IgG control or rat anti-CCR3 (αCCR3) **(F)** or in WT compared with CCR3 deficient mice **(I)** at indicated time points post-ip infection with 50 *Bm*L3. Data from individual mice with median and interquartile range are plotted. Significant differences between naïve or infected WT controls and experimental groups at a given time point is assessed by Mann-Whitney or Kruskal-Wallis + Dunn’s tests (>2 groups). Data is plotted is either pooled from 2 individual experiments per time-point or from individual experiments with groups of 4–6 mice per group per time-point.

Previous studies have highlighted a role of tissue eosinophilia as an important factor in immunity to chronic filarial infections[3, 5]. We examined the eosinophil dependency of immune control of *B*. *malayi* infections in non-permissive BALB/c mice. In this model, ~90% of infectious larvae do not survive to develop into adult nematodes (+35dpi) and sterile cure is apparent in most mice before fecund infections establish (+84dpi, at a time point when female *B*. *malayi* are releasing microfilariae; mf). Utilizing mice with disrupted regulation of the GATA-1 gene (ΔdblGATA^-/-^), essential for the development of eosinophils from bone marrow precursors[28], the impact of eosinophil deficiency could be evaluated. Confirming deficiency, SigLecF^+^ tissue eosinophilia was absent in ΔdblGATA^-/-^ mice, +14dpi, compared with WT mice (Fig 1A & 1B). The impact of ablating tissue eosinophilia in ΔdblGATA^-/-^ mice was an increased susceptibility to developing, immature larvae *B*. *malayi* infection, +14dpi, and permissiveness to chronic adult *B*. *malayi* infections, +84dpi (Fig 1C). Murine circulating eosinophils express the chemokine receptor CCR3 and respond to CCR3-specific chemokines to migrate to tissue sites of inflammation. We utilized a CCR3 neutralising antibody [29] to temporarily deplete CCR3^+^ cells in WT mice prior to infection. Tissue eosinophilia and *B*. *malayi* development was tracked over the first 35 days of infection. A single treatment of αCCR3 was sufficient to reduce >95% infection-site tissue eosinophilia (Fig 1D & 1E) and this was concomitant with increased *Brugia* survival +6dpi (Fig 1F). By +14dpi eosinophilia had resumed comparable to IgG control treated WT mice (Fig 1D & 1E). The resumption of eosinophilia was associated with rapid decline in susceptibility, where levels of *B*. *malayi* larvae were not different from untreated, infected WT mice (Fig 1F). We further addressed CCR3-dependency of tissue eosinophilia and impact on immunity to *B*. *malayi* by using CCR3 deficient mice where steady state eosinophils in peripheral circulation are maintained but their CCR3-dependent tissue recruitment is ablated[30]. CCR3 deficiency rendered a profound, sustained impairment in tissue eosinophilia throughout the course of *B*. *malayi* infection (Fig 1G & 1H). CCR3 deficiency rendered mice susceptible to the development of chronic *B*. *malayi* adult infections (Fig 1I), including permissiveness to fecund infections able to complete the filarial parasite life cycle +84dpi (S1 Fig).

### In situ proliferation and alternative activation of Mϕ occurs coincident with eosinophilia

**Fig 2:**
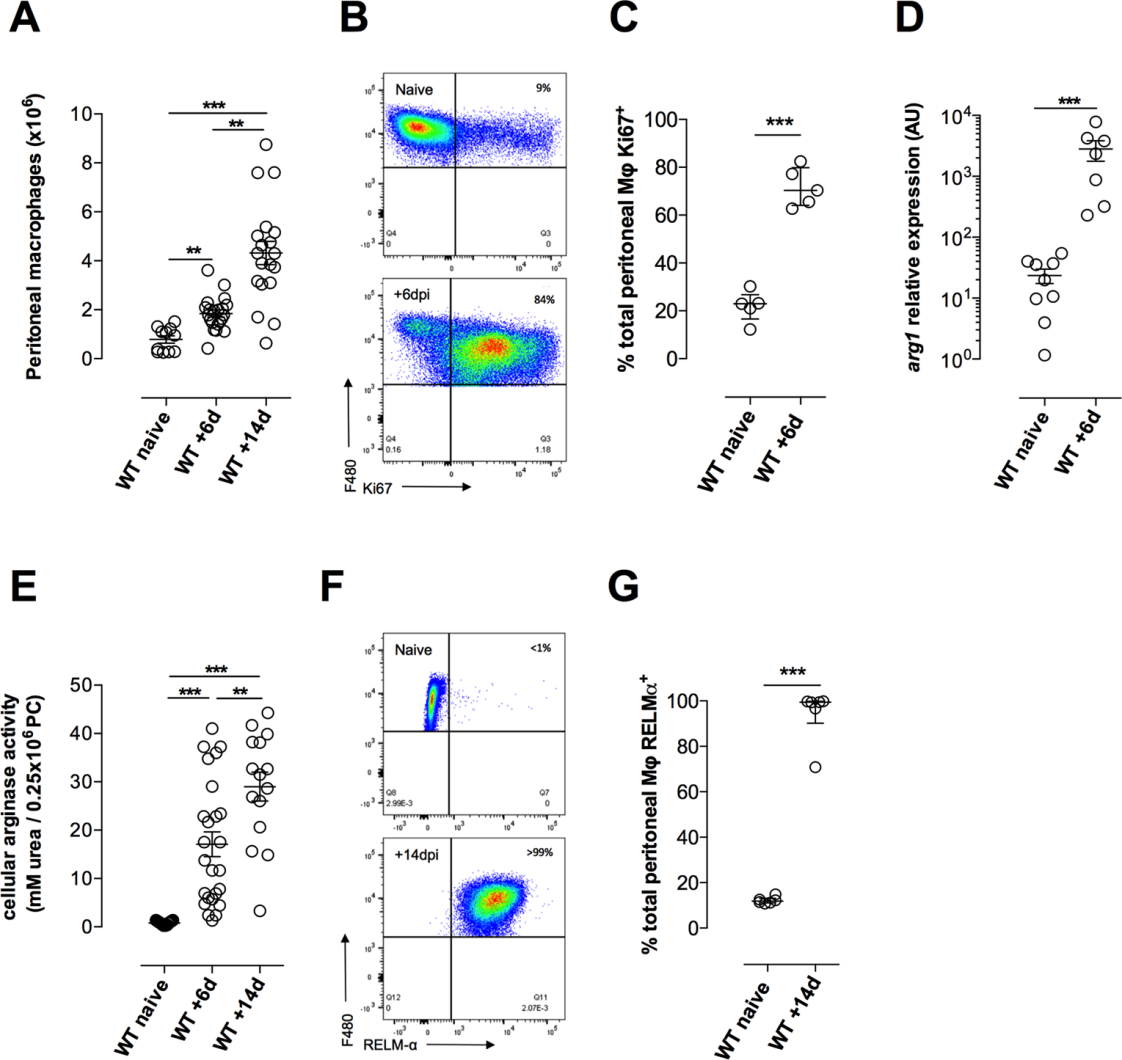
*In situ* proliferation of macrophages with an alternatively-activated phenotype develop at the site of *B*. *malayi* infection. Expansion of F4/80 peritoneal Mϕ **(A)**, F4/80 peritoneal Mϕ expression levels of Ki67 **(B,C)**, peritoneal cell (PC) *arg1* expression **(D)** PC arginase activity **(E)** and F4/80 peritoneal Mϕ expression levels of RELMα **(F,G)** in WT BALB/c mice at indicated time points post-infection with 50 *Bm*L3. Data from individual mice with median and interquartile range are plotted. Significant differences between naïve or infected WT groups at a given time point is assessed by Mann-Whitney or Kruskal-Wallis + Dunn’s tests (>2 groups). Data is plotted is either pooled from 2–3 individual experiments per time-point or from individual experiments with groups of 4–6 mice per group per time-point.

Expansion of Mϕ has been described at serous cavities of filarial nematode infection, in a mechanism of *in situ* proliferation[26, 27]. At the infection site, time-dependent expansions of Mϕ were evident from +6–14 dpi (Fig 2A). We examined proliferation and activation status of infection-site Mϕ. By Ki67 intracellular staining we determined the majority of Mϕ expanded +6dpi were in an active proliferation cycle (median 70.4%, range 62–84%) (Fig 2B & 2C). By measuring the AAMϕ product, arginase, we defined that *arg1* transcripts and enzymatic activity within peritoneal cells (PC) from *B*. *malayi* (*Bm*)L3 primary infections were significantly enhanced compared with naïve mice (Fig 2D & 2E). Elevated Mϕ-specific *arg1* transcripts during infection were confirmed following purification from PC by FACS (S2 Fig). By intracellular staining for resistin-like molecule-alpha (RELMα), a helminth-activated Mϕ product[9, 26], we discerned high levels of RELMα protein expression in the expanded pool of peritoneal Mϕ +14d following *Bm*L3 infection (Fig 2F & 2G).

### Development of arginase-producing AAMϕ post-*B*. *malayi* infection requires adaptive-immune IL-4/IL-4Rα signalling but not eosinophilia

**Fig 3:**
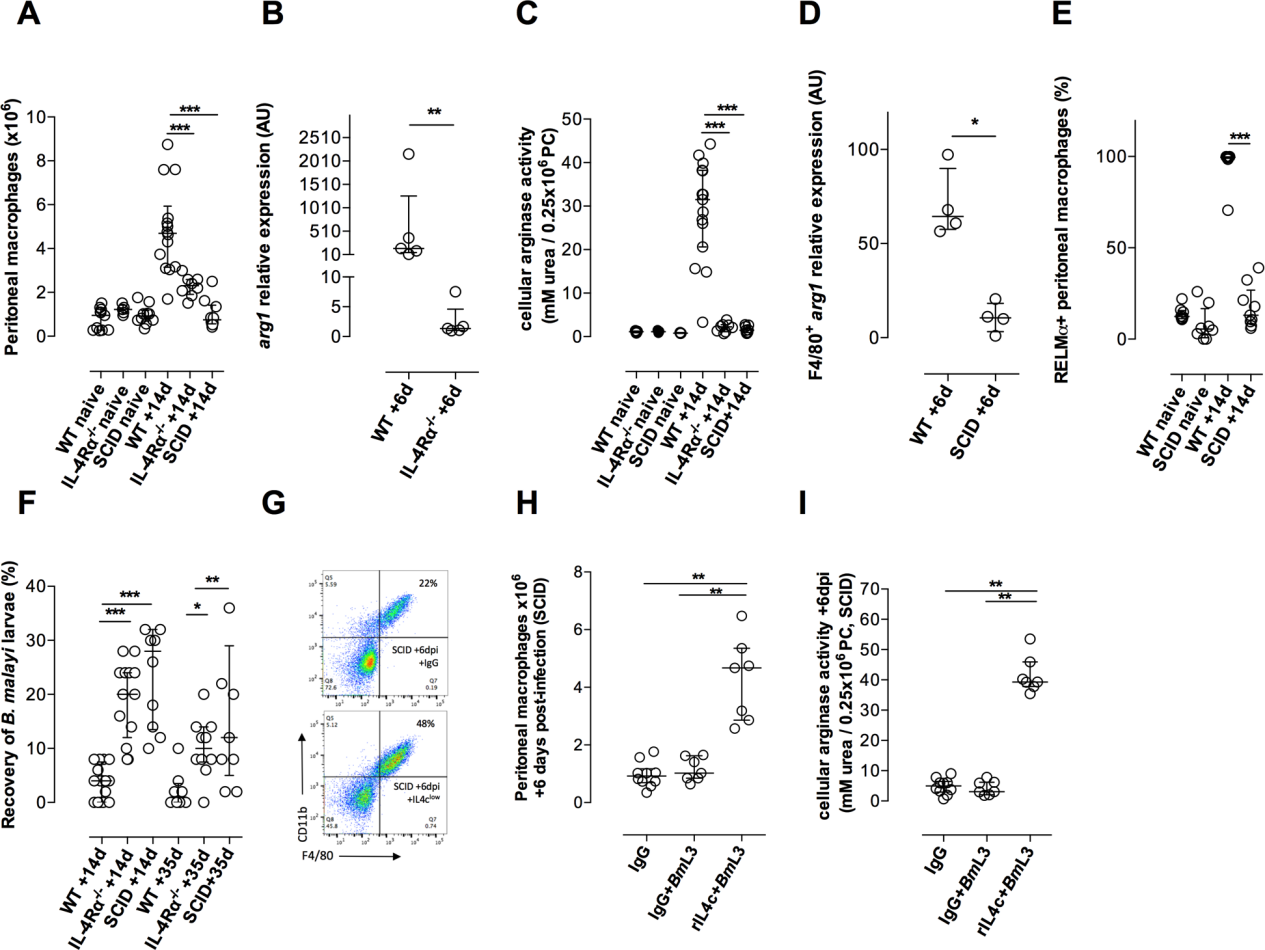
Development of AAMϕ in response to *B*. *malayi* infection requires adaptive-immune IL-4/IL-4Rα signalling and is associated with resistance to adult parasite establishment. Expansion of peritoneal Mϕ **(A)** peritoneal cell (PC) *arg1* expression **(B)** PC arginase activity **(C)** peritoneal Mϕ *arg1* expression **(D)** Mϕ RELMα expression **(E)** and recovery of *B*. *malayi* **(F)** at indicated time points post-infection with 50 *Bm*L3 in BALB/c WT, IL-4Rα^-/-^ or SCID mice or in naïve controls. Expansion of peritoneal Mϕ **(G,H)** or PC arginase activity **(I)** in BALB/c SCID mice +6d post-treatment with recombinant murine IL-4+rat anti-mouse IL-4 monoclonal antibody complex (rIL-4c) or rat IgG control ip treatments with or without infection with 50 *Bm*L3. Data from individual mice with median and interquartile range are plotted. Significant differences between groups assessed by Mann-Whitney or Kruskal-Wallis + Dunn’s post-hoc tests (>2 groups). Data is from an individual experiment or pooled from 2–3 experiments per time-point using groups of 4–6 mice per group / time-point.

Interleukin(IL)-4 and IL-13 can induce alternative activation of Mϕ populations in diverse tissue sites during helminth infections via the IL-4 receptor (IL-4R)[9, 20, 26, 27]. Intra-peritoneal infections with *Brugia* larvae induce polarized Th2 responses[31] and we recorded increased splenic Th2 immune responses +6 dpi with *Bm*L3 (S3 Fig). However, because IL-4R-independent AAMϕ differentiation has also been demonstrated in helminth infections[26, 32], we examined Mϕ development in either Severe-combined (SCID; no functional T or B cells) or IL-4Rα deficient (IL-4/IL-13 non-responsive) BALB/c mice. Compared with WT mice, Mϕ expansions and Mϕ arginase expression, arginase activity and RELMα production was significantly hindered from SCID or IL-4Rα^-/-^ mice +14-35dpi (Fig 3A–3E). Both severe-combined and IL-4Rα-specific deficiencies rendered mice susceptible to chronic *B*. *malayi* adult-stage infections at +35dpi with significant differences apparent in the control of larval establishment from +14dpi (Fig 3F). We delivered exogenous murine recombinant (r)IL-4, as a long-acting formulation (complexed to rat anti-IL-4) into the peritonea of BALB/c SCID mice and determined that rIL-4 delivery +*Bm*L3 infection was sufficient to recapitulate Mϕ expansions and elevate arginase production in severe-combined immunodeficiency (Fig 3G–3I and S4 Fig). Combined, this data indicates that provision of an adaptive immune IL-4:IL-4Rα ligating signal transduced either directly within peritoneal Mϕ or via non-lymphocyte lineages intact in SCID mice, is sufficient to support the development of the AAMϕ phenotype induced by *B*. *malayi* infection.

**Fig 4:**
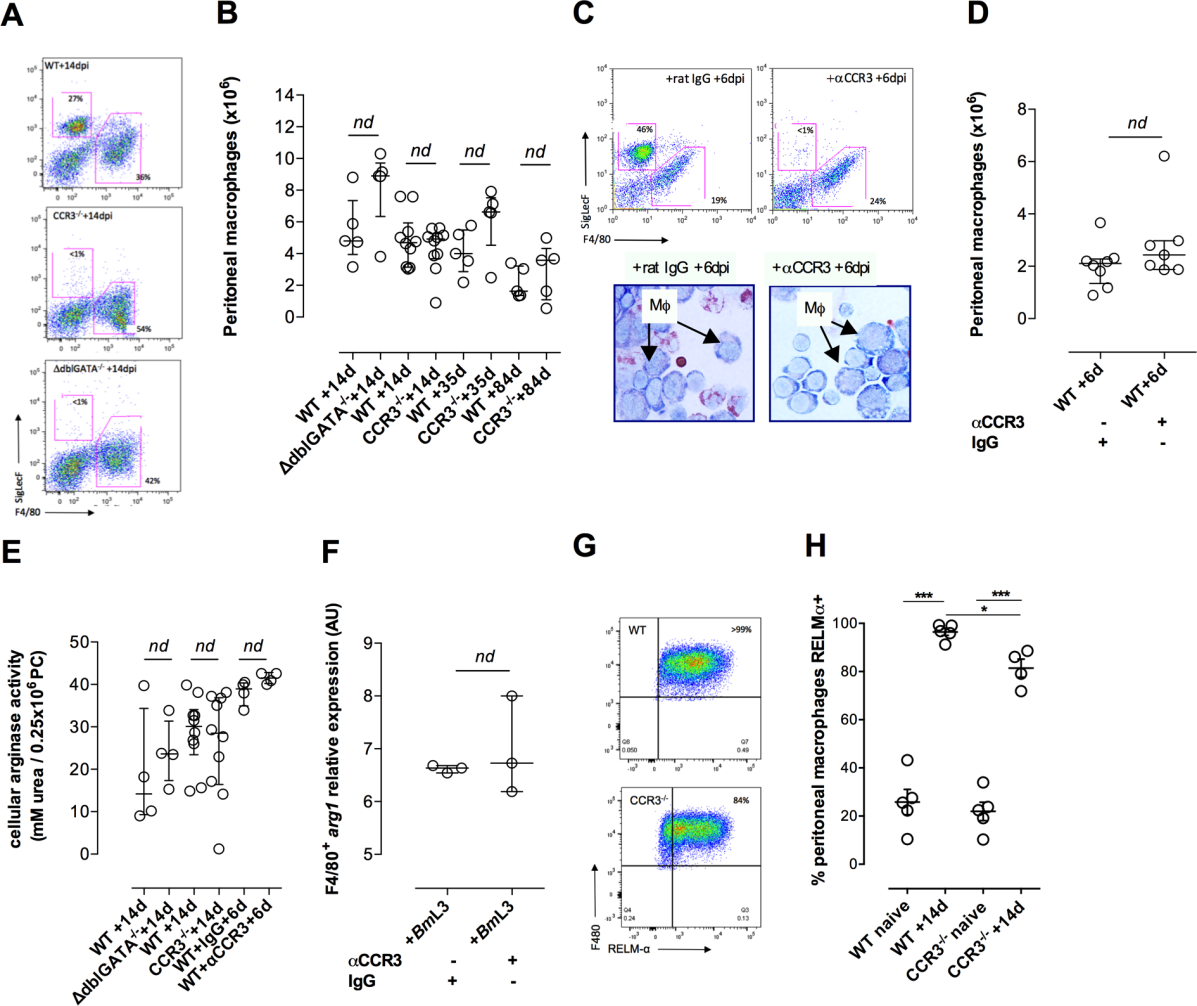
Eosinophilia does not impact on expansion of arginase-expressing AAMϕ but augments RELMα production. Expansion of peritoneal Mϕ in BALB/c WT, ΔdblGATA deficient mice or CCR3 deficient mice **(A,B)** or in WT mice treated ip with rat IgG control or rat αCCR3 **(C,D)** at indicated time points post-ip infection with 50 *Bm*L3. Arginase activity in PC cells from BALB/c WT, ΔdblGATA deficient or CCR3 deficient mice, WT mice treated ip with rat IgG control or rat αCCR3 **(E)** and expression of *arg1* in purified Mϕ from WT mice treated ip with IgG control or αCCR3 **(F)** at indicated time points post-ip infection with 50 *Bm*L3. F4/80 peritoneal Mϕ expression levels of RELMα in BALB/c WT or CCR3^-/-^ mice at +14 day post-infection with 50 *Bm*L3 **(G,H)**. Data from individual mice with median and interquartile range are plotted. Significant differences between groups assessed by Mann-Whitney or Kruskal-Wallis + Dunn’s tests (>2 groups). Data is from an individual experiment or pooled from 2–3 individual experiments per time-point using groups of 3–6 mice per group / time-point.

Eosinophils have diverse immune-regulatory functions and can also influence AAMϕ activation, potentially by provision of IL-4/IL-13 cytokine delivery[9, 33–35]. We assessed whether deficiency in tissue eosinophilia affected the development of AAMϕ post-*Bm*L3 infection. The impaired eosinophilia evident at the infection site using either eosinophil-lineage depleted or CCR3^-/-^ mice did not impinge on Mϕ expansions post-infection (Fig 4A & 4B). Further, CCR3-deficiency did not affect initial Mϕ expansions post-infection or their chronic maintenance +35dpi to +84dpi (Fig 4B). Temporary antibody depletion of CCR3 cells similarly did not impact on initial peritoneal Mϕ expansions +6dpi (Fig 4C & 4D). Arginase production within the infection-expanded Mϕ pool was not significantly different in tissue BALB/c eosinophilia-deficient mice compared with WT, adjudged by arginase activity or Mϕ-specific *arg1* transcripts (Fig 4E & 4F). Infection of CCR3^-/-^ mice also induced a high-level induction of RELMα expression in expanded peritoneal Mϕ (Fig 4G & 4H). However, the expression levels of RELMα were subtly, yet significantly, modified compared with WT mice, indicating a degree of eosinophil ‘help’ in the full induction of RELMα within AAMϕ post-*Bm*L3 infection (Fig 4G & 4H). These data indicate that whilst adaptive immune provision of an IL-4Rα ligating signal is critical for AAMϕ development during *B*. *malayi* infection, eosinophilia is not essential for arginase production or AAMϕ expansion.

### *‘Bm*L3AAMϕ*’* are required for the immune control of *B*. *malayi* larvae

**Fig 5:**
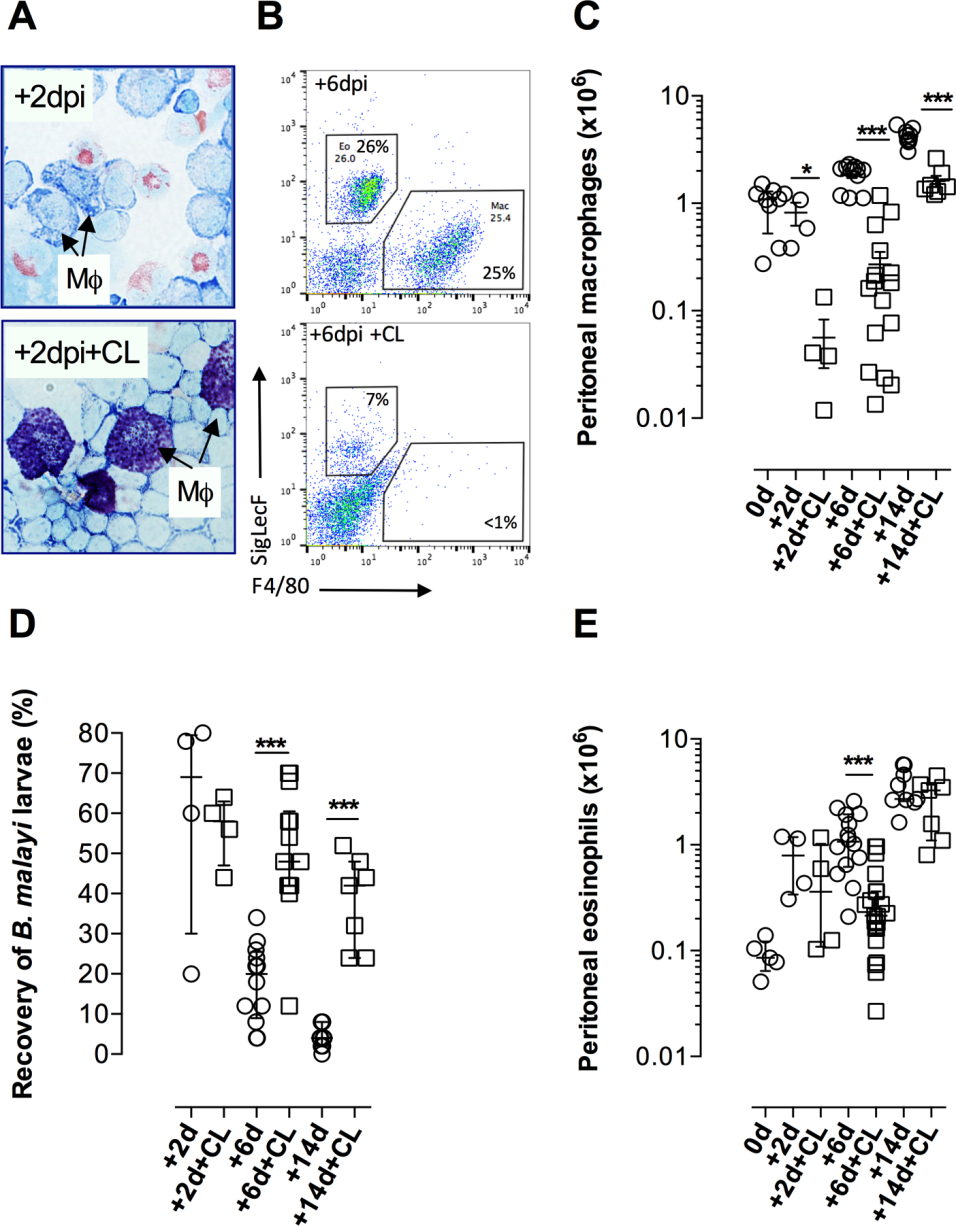
Temporal ablation of peritoneal Mϕ enhances survival of *B*. *malayi* larvae coincident with impaired tissue eosinophilia. Cytospins of peritoneal cells with macrophages (Mϕ) indicated **(A)**, quantification of macrophages and eosinophils **(B,C,E)** and recovery of *B*. *malayi* larvae **(D)** at indicated time points post-infection with 50 *B*. *malayi* L3 with or without prior treatment with clodronate liposomes (CL) in BALB/c WT mice or naïve controls (d0). Data from individual mice with median and interquartile range are plotted. Significant differences between groups per time point assessed by Mann-Whitney. Data is from an individual experiment or pooled from 2–3 individual experiments per time-point using groups of 4–6 mice per group / time-point.

We addressed the functional relevance of the expanded pool of tissue AAMϕ post-*Bm*L*3* infection, subsequently termed, “*Bm*L3AAMϕ”, in the immune response to *B*. *malayi* by ablating resident phagocytes by ip administration of clodronate liposomes (CL), prior to infection. Success of resident Mϕ ablations were confirmed by observing apoptotic Mϕ cells in cytospin preparations and >90% reductions in peritoneal F4/80^+^ Mϕ numbers in infected WT mice, three days after injection of CL and +2dpi (Fig 5A & 5B). CL administration suppressed the initial expansion of *Bm*L3AAMϕ, with Mϕ numbers remaining <90% of infection controls at +6dpi before recovering to 30–40% of WT controls by +14dpi (Fig 5B & 5C). The impact of CL treatment and concomitant temporal depletion of AAMϕ was a significant increase in *B*. *malayi* larval survival (Fig 5D). CL treatment did not modify immune priming of the larvicidal Th2 adaptive immune response, as post-CL Th2 splenocyte responses to larval antigen remained intact (S5 Fig). However, peritoneal eosinophilia was temporarily, yet significantly, impacted by CL treatment at +6 dpi (approx. 90% reduction in eosinophilia; Fig 5E). In follow up assessments, as well as the temporal detrimental impact on Mϕ and eosinophilia, we discerned that the ip administration route of CL also impacted both on circulating monocytes in WT naïve BALB/c mice (S6 Fig), as well as partial increases in numbers of neutrophils and partial decreases in peritoneal B cells at the infection site in WT mice at +6dpi (S6 Fig).

### *Bm*L3AAMϕ are not directly larvicidal but are necessary to sustain a larvicidal tissue eosinophilia

**Fig 6:**
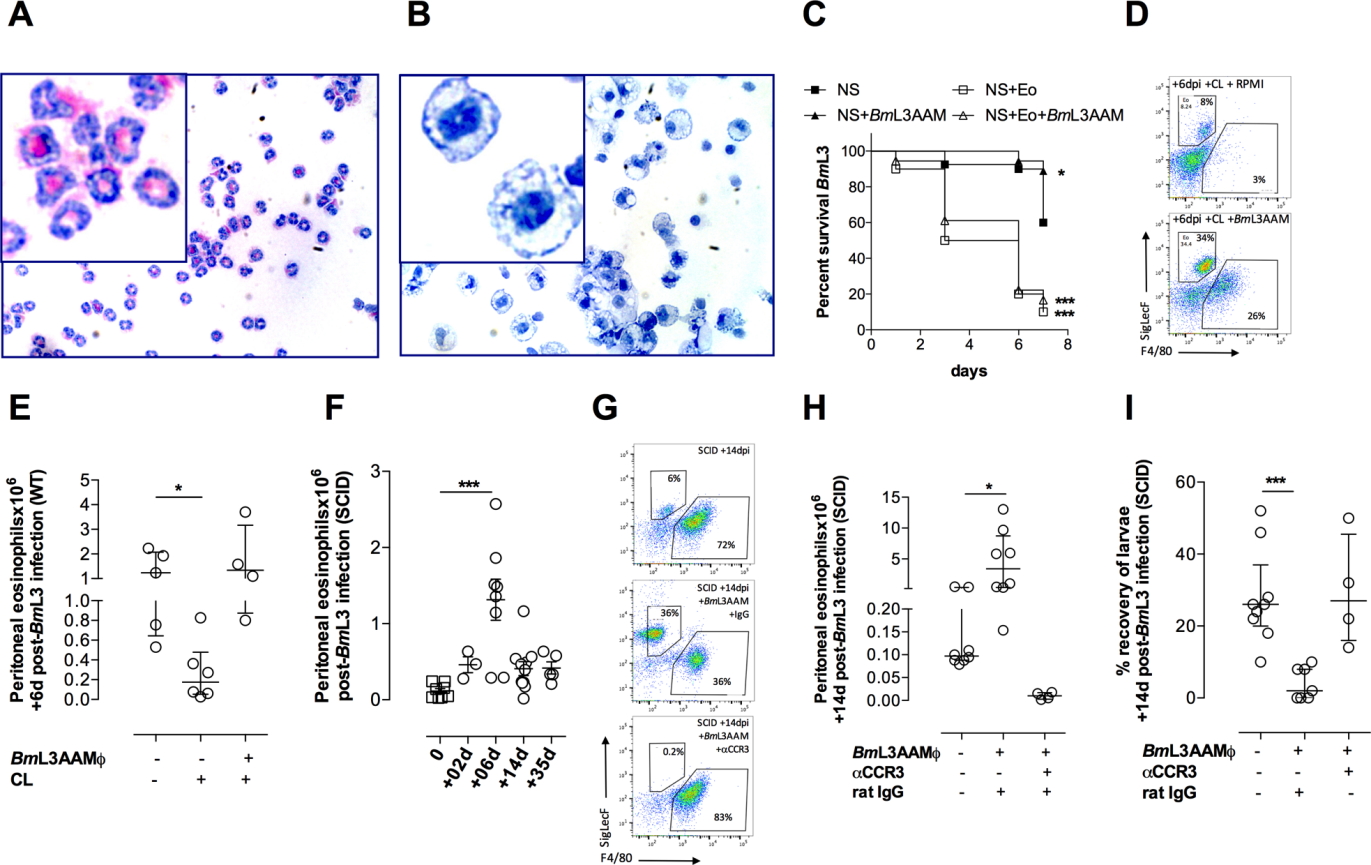
*Bm*L3AAMϕ are necessary to sustain a larvicidal tissue eosinophilia. Cytospins of FACS-sorted BALB/c WT peritoneal SigLecF+ eosinophils **(A)** or *Bm*L3AAMϕ **(B)** +14 days post-infection with 50 *Bm*L3. Survival analysis of *Bm*L3 **(C)** throughout 7-days culture with normal mouse serum (NS) or co-cultured with 10^6^ FACS-sorted eosinophils (Eo), 10^6^ *Bm*L3AAMϕ or combinations of Eo+*Bm*L3AAMϕ, (cells sourced as for A,B). Data is pooled from two individual experiments evaluating motility of 10 *Bm*L3 per condition. Significance of Kaplein-Meir survival analysis *vs* NS serum control is indicated per condition. Eosinophilia **(D,E)** +6dpi with 50 *Bm*L3 ip in BALB/c WT mice pre-treated with clodronate liposomes (CL) ip +/- adoptive transfer of 0.75x10^6^ *Bm*L3AAMϕ ip (cells sourced as for B). Time course of peritoneal eosinophilia in BALB/c SCID mice at indicated time points post infection with 50 *Bm*L3 ip **(F)**. Peritoneal eosinophilia **(G,H)** or recovery of *B*. *malayi* larvae **(I)** at +14 days post-infection with 50 *Bm*L3 in BALB/c SCID and E) and pre-treatment with either rat IgG or rat αCCR3 antibody. Data from individual mice with median and interquartile range are plotted. Significant differences between groups assessed by Kruskal-Wallis + Dunn’s tests. Data is from an individual experiment or pooled from 2–3 individual experiments per time-point using groups of 4–6 mice per group / time-point.

**Fig 7:**
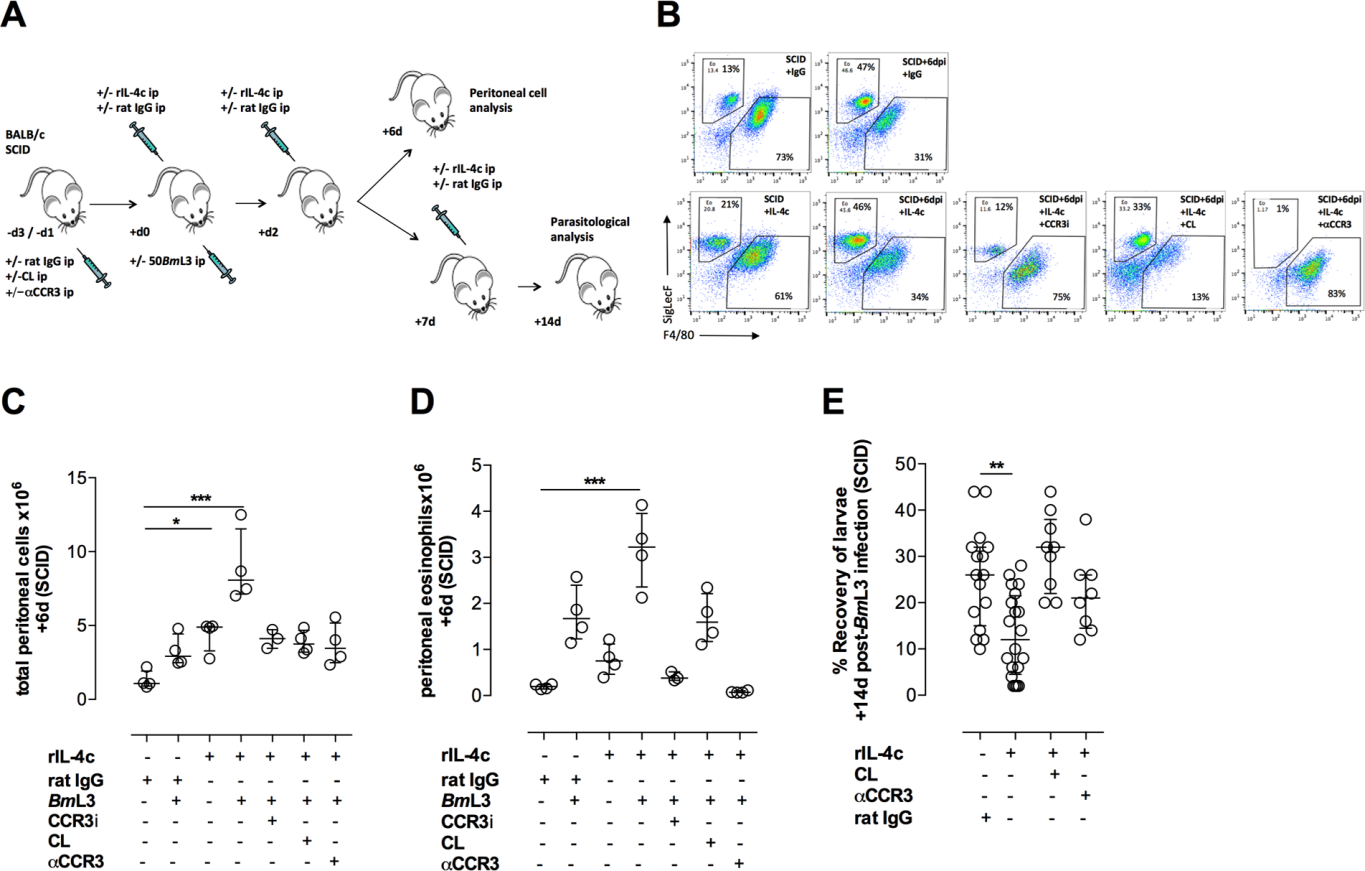
Exogenous IL-4 bolsters CCR3-dependent eosinophilia and the eosinophilic larvicidal response in SCID mice via *Bm*L3AAMϕ development. Schematic of experimental approach **(A).** Flow cytometric assessments of F4/80 Mϕ or SigLecF eosinophil proportions **(B),** total peritoneal cell number **(C),** eosinophil number **(D)** at +6dpi or larval parasite recoveries **(E)** at +14dpi in BALB/c SCID mice pre-treated ip with either rat IgG, clodronate liposomes (CL) or rat αCCR3 ip, prior to infection with 50 *Bm*L3 and/or up to three doses of rat IgG (25μg), IL-4c (1μg rIL-4 complexed to 5μg rat anti-IL-4) delivered 0d, +2d, +/- +7d with or without daily oral dosing with CCR3 inhibitor. Data from individual mice with median and interquartile range are plotted. Significant differences between test groups and appropriate rat IgG treated controls assessed by Kruskal-Wallis + Dunn’s tests. Data is representative of two individual experiments **(B,C)** or pooled from 2–3 individual experiments using groups of 3–6 mice per group / time-point.

Because of the pleiotropic effects of CL administration on multiple cell types both local and distal to the site of infection, we sought to isolate the relative roles of *Bm*L3AAMϕ and eosinophilia in mediating immunity to *B*. *malayi*. To directly test the relative requirements of peritoneal eosinophils recruited by *Bm*L3 infection or *Bm*L3-activated AAMϕ, we performed *in vitro* motility assays whereby groups of 10 *Bm*L3 were co-cultured with either 10^6^ purified peritoneal recruited eosinophils, 10^6^ *Bm*L3AAMϕ or combination of both cell types, sourced from *B*. *malayi* WT infections by FACS (Fig 6A & 6B). After tracking motility +7d, peritoneal eosinophil cultures contained 10% motile larvae compared with 60% in serum-only cultures (Fig 5C). This reduction in motility in the presence of eosinophils was manifest with or without co-culture with *Bm*L3AAMϕ. Surprisingly, *Bm*L3AAMϕ-only cultures potentiated the motile phenotype of *Bm*L3 +7d compared with serum only cultures (90% *vs* 60% motile *Bm*L3), indicating that fully polarised, WT *Bm*L3AAMϕ, producing high levels of arginase and RELMα protein are not directly larvicidal *in vitro*. We next examined whether *Bm*L3AAMϕ were necessary in CCR3-dependent tissue eosinophilia during infection. We added back 0.75x10^6^ purified *Bm*L3AAMϕ from BALB/c WT infections, +3d following CL-treatment and at the point of infection in BALB/c WT mice. Establishment of adoptively transferred *Bm*L3AAMϕ was confirmed by increased F4/80^+^ Mϕ numbers compared with CL treated controls (Fig 6D & S7 Fig). Restoration of *Bm*L3AAMϕ coincided with a vigorous eosinophilia, comparable to infected WT controls (Fig 6D & 6E). To measure subsequent impact on larval survival, we utilised BALB/c SCID mice in which AAMϕ fail to develop and chronic adult infections establish [36]. We observed a transient spike in tissue eosinophilia in BALB/c SCID mice at +6dpi where peritoneal eosinophils had dissipated by +14dpi (Fig 6F). However, following adoptive transfer of *Bm*L3AAMϕ, tissue eosinophilia was sustained at a density comparable to WT infections in SCID recipients at +14dpi (Fig 6G & 6H). Engraftment of transferred *Bm*L3AAMϕ was confirmed both by increased Mϕ number and increased arginase activity in SCID recipients (S7 Fig). Adoptive transfer of *Bm*L3AAMϕ rendered SCID mice resistant to *B*. *malayi* infection and was dependent on CCR3^+^ cell recruitment in SCID recipients because αCCR3 treatment effectively nullified the sustained eosinophilia in *Bm*L3AAMϕ SCID recipients and reversed the resistant phenotype in controlling larval establishment (Fig 6G–6I). Because rIL-4, in combination with *B*. *malayi* infection, could recapitulate the WT *Bm*L3AAMϕ phenotype in SCID mice (Fig 2), we examined the impact of exogenous rIL-4 treatment on tissue eosinophilia in SCID deficiency (Fig 7A). We determined eosinophilia was dependent on dose of rIL-4 delivered, with low but not high levels of rIL-4 mediating elevated peritoneal eosinophils in isolation (S4 Fig, Fig 7B, 7C and 7D). Tissue eosinophilia was significantly bolstered following infection coincident with rIL4 treatment (Fig 7B, 7C and 7D). Using an oral CCR3 inhibitor[37], tissue eosinophilia could be blocked in the face of rIL-4 treatments and *Bm*L3 infection (Fig 7B & 7D). Together these data indicate that ligation of IL-4Rα and subsequent *Bm*L3AAMϕ development augments tissue eosinophilia via CCR3 chemotaxis during the adaptive immune response to infection. In support of this, via transcript analysis of peritoneal cells we identified a significant reduction in CCL11 (eotaxin 1) expression in IL-4Rα deficient mice 6 days after infection with *Bm*L3 (S8 Fig). Because rIL-4 delivery can induce pleiotropic effects on IL-4 responsive cell types, which could influence tissue eosinophilia, we addressed the specificity of *Bm*L3AAMϕ by ablating Mϕ prior to rIL-4 delivery and infection. Following depletions of peritoneal Mϕ mediated by CL, tissue eosinophilia was not significantly elevated +6dpi in rIL-4 treated SCID mice (Fig 7B & 7D). The parasitological outcome of IL-4/IL-4Rα activation of *Bm*L3AAMϕ and CCR3-dependent tissue eosinophilia was a significant reduction in *B*. *malayi* larvae in SCID mice +14dpi (Fig 7E). However, temporal ablations of peritoneal Mϕ or CCR3+ eosinophils (by CL or αCCR3, respectively) nullified the effect of rIL-4 in larval killing (Fig 7E). These data define a role for Th2 adaptive immune induced AAMϕ as important regulators of filaricidal tissue eosinophilia via CCR3-mediated chemotaxis.

## Discussion

Our data demarcates the relative contributions of the hallmark Th2-associated cell types, eosinophils and AAMϕ, in filarial helminth immunity. Our data reveals a mechanism whereby eosinophil-dependent immunity to the filarial helminth, *B*. *malayi*, is locally coordinated by an *in situ* proliferating pool of Mϕ, activated by combination of ligation of IL-4Rα and parasite infection. Mϕ alternative activation and polarisation is a consistent feature of helminth infection[17], yet a defined role of this cell phenotype in immunity to worm infection has remained elusive. AAMϕ-mediated immunity has been demonstrated in situations of Th2 memory and parasite-specific antibody leading to control of gut nematode larvae during secondary infections. In these challenge infection experiments, larval trapping of *H*. *polygyrus bakeri* within the gut mucosa[32, 38] or *N*. *brasiliensis* within skin[39] is impaired if inflammatory AAMϕ recruitment to infection sites are blocked. A direct mechanism of worm attrition by AAMϕ-released factors within mucosal larval granulomas, including arginase, has been identified, following FcR-antibody-dependent alternative activation[32] [40]. Further *in vitro* evidence supports corroboration between AAMϕ and neutrophil granulocytes in larvicidal activity against the human gut nematode, *Strongyloides stercoralis* [41].

Our data demonstrates a unique mode of action of AAMϕ-orchestrated, eosinophilic immunity to filarial nematodes at a non-barrier site of infection. Firstly, we define that a *B*. *malayi* larvicidal response can be induced by targeting IL-4R in antibody-deficient mice, suggesting ADCC is not an absolute requirement for filarial larval killing. However, parasite-specific antibody may bolster worm killing following FcR engagement on Mϕ, as we observed more profound larvicidal effects upon transfer of +14 day *Bm*L3AAMϕ generated from WT infection (where anti-parasite antibody would presumably be bound to Mϕ FcR) compared with *in vivo* IL-4R ligation and *Bm*L3AAMϕ development within SCID mice. Secondly, we demonstrate conservation of arginase production in AAMϕ during eosinophil deficiency, which are yet insufficient to prevent the establishment of chronic adult filarial infection. Thirdly, *in vitro* co-cultures show no deleterious effect of *Bm*L3AAMϕ in isolation on *Bm*L3 motility. These differences may highlight fundamental distinctions in immune-effector processes during primary infection between AAMϕ subsets proliferating from local Mϕ populations in the serous cavities and those recruited from inflammatory blood monocytes via CCR2 at barrier sites of challenge infection[39, 42]. Potentially, it may also indicate inherent differences in susceptibility of filarial *vs* gut nematode larvae to Mϕ-specific secreted products such as arginase.

We demonstrate that optimum peritoneal Mϕ expansion and alternative activation is IL-4Rα dependent during *B*. *malayi* larval infection and further show that this phenotype can develop in the absence of functional adaptive lymphocyte lineages via exogenous delivery of IL-4. One obvious mechanism for this polarization and proliferation is direct ligation of resident peritoneal macrophage IL-4Rα by IL-4/13 in combination with the complement factor C1q[27, 43]. However, because Mϕ alternative activation can occur independently of IL4R via FcR ligation [32] or other polarising signals such as IL-33 [35], we do not rule out a role for Mϕ alternative activation signals being triggered by non-lymphocyte, IL-4 responsive cell types in our infection system.

Cross-talk between granulocyte populations and AAMϕ mediates diverse functional outcomes, including immunity[40]^,^[39], immunomodulation[9], and maintenance of glucose homeostasis[33, 44]. In certain situations, granulocytes are important cellular sources of polarising signals instructing macrophage alternative-activation. Beyond arginase, RELMα and Ym-1 are abundantly expressed molecules in helminth-activated Mϕ[17]. We detected a subtle impact of deficiency in tissue eosinophilia in modifying the level of RELMα expression within AAMϕ, supporting earlier work in *L*. *sigmodontis* infected eosinophil deficient mice[35]. Further, Ym-1 production is demonstrably impaired in AAMϕ in response to *L*. *sigmodontis* in the absence of eosinophils[35]. Our *in vitro* assays indicate that arginase- and RELMα-producing WT *Bm*L3AAMϕ do not affect larval viability in isolation and our adoptive transfer experiments into SCID recipients further indicate that arginase- and RELMα-producing WT *Bm*L3AAMϕ do not affect *B*. *malayi* larval survival if CCR3 expressing cells and eosinophilia is effectively ablated. Therefore, we conclude that whilst eosinophil ‘help’ may contribute to the IL-4Rα-dependent polarisation of *Bm*L3AAMϕ, we find no evidence from these experiments supporting a direct larvicidal mode of action of AAMϕ *in vitro* or *in vivo* against *B*. *malayi*, using the BALB/c ip infection model.

GATA deficiency has latterly defined to disrupt basophil haematopoesis as well as ablating mature eoinophils [45] whilst mast cells are unaffected in ΔdblGATA1^-/-^ mice [46] and neither is their recruitment to inflammed tissue compromised in CCR3 deficiency [30]. Murine basophils are recruited to tissue niches in a CCR3-independent mechanism and do not express CCR3 [47, 48]. Thus, we carefully selected complementary systems (ΔdblGATA deficiency, CCR3 deficiency and CCR3 depleting antibody) to selectively target eosinophils whilst controlling for potential ‘off-target’ impact on basophilia or mastocytosis during peritoneal *Brugia malayi* larval infection.

Recent studies in our laboratories have defined that origin of local tissue macrophage populations varies with age, gender, strain and infection status. Whilst embryonic self renewing macrophages predominate in young mice, in aged mice, bone marrow derived monocyte precursors continually seed the peritoneum during steady state to establish into long-lived self-renewing macrophages of similar tissue phenotype[49]. Interestingly, during filarial infection of the pleural cavity of BALB/c mice, the relative proportions CCR2-monocyte recruited macrophages increases relative to resident proliferating populations as chronicity of infection progresses[50]. Therefore, an increasing heterogeneity in local macrophage populations during infection may influence magnitude of eosinophil granulocyte influx.

In the absence of adaptive IL-4/IL-13 signalling, a transient spike in innate immune tissue eosinophilia is apparent during initial *B*. *malayi* infection, at day 6, which dissipates on or before day 14. This kinetic has also been observed in experimental *Brugia* infections using SCID mice on a C57Bl/6 background[51]. Our data indicates that expansion and alternative activation of Mϕ populations within the serous cavity from 6 days post-infection is critical to amplify tissue eosinophilia to drive immunological resistance during filarial infection. Previous studies have demonstrated a role for IL-4 responsive AAMϕ in positively regulating eosinophil trafficking during situations of Th2 inflammation in the lung or gut[47, 52]. In our *B*. *malayi* BALB/c infection model, CCR3-mediated chemotaxis was fundamental in the AAMϕ-dependent eosinophilia during *Brugia* larval infection as blocking CCR3 signalling ablated eosinophil recruitment to the peritoneum. Post-infection, the CCR3 ligand, CCL11, was upregulated at the transcript level in peritoneal cells and relative transcripts were significantly impaired in *Bm*L3-infected IL-4Rα^-/-^ mice. In previous RNA-seq analysis of AAMϕ polarised by *Brugia* adult implantations into BALB/c mice, the CCR3 ligands, CCL8 and CCL24 have been identified as upregulated transcripts[53]. It is therefore likely that a repertoire of CCR3 ligands are produced by the resident pool of Mϕ, possibly with distinct kinetic expression profiles, as they undergo proliferation and alternative activation during the first two weeks of infection. Because, as well as eosinophils, Mϕ comprise a major cell type in granulomas formed around entrapped filarial larvae[54], we suggest that AAMϕ may focally recruit eosinophils to the nematode cuticle and orchestrate eosinophilic larvicidal granuloma formation *in vivo*.

Medically and veterinary important filarial parasites establish in diverse, non-barrier tissues including the peritoneum. Thus, local Mϕ Th2-induced proliferation and alternative-activation at these sites of infection may orchestrate diverse eosinophil-associated outcomes in filariasis, including sterilising immunity, immune control of circulating mf and acute immunopathologies induced following the death of filariae in parasitized tissues.

## Materials and methods

### *B*. *malayi* experimental infections

IL-4Rα^-/-^, CCR3^-/-^ or dblΔGATA^-/-^ mice (BALB/c) were purchased from Jax Labs USA. WT and SCID BALB/c mice were purchased from Harlan UK. Rodents were maintained in SPF conditions at the University of Liverpool Biological Services Unit. Infectious stage *B*. *malayi* L3 were propagated as previously described[36]. Male mice 6–10 weeks of age were infected with 50 *Bm*L3 i.p. and infections maintained between +6-84d. Motile *B*. *malayi* parasites and exudate cells were recovered by peritoneal lavage at necropsy and enumerated by microscopy. All experiments on animals were approved by the ethical committees of the University of Liverpool and LSTM, and were conducted according to Home Office Legislation and ARRIVE guidelines.

### Flow cytometry

Single cell suspensions were prepared in FACS buffer (PBS+0.5%BSA+2mMEDTA). Fc receptors were blocked with αCD16/32 (eBioscience). Live/dead cell differentiation was undertaken with fixable viability dye efluor 450 as per manufacturer’s instructions (eBioscience). Cell staining was undertaken utilising specific labelled anti-mouse antibodies or their matched isotype controls using a fluorescence-minus-one method. Intracellular staining was done following permeabilisation buffer treatment (eBioscience). using a zenon Alexa Fluor 488 Rabbit IgG labelling kit as per manufacturer’s instructions (Invitrogen). All multi-labelled cell samples were subsequently acquired using a BD LSR II flow cytometer (BD Bioscience) and analysed on FloJo Software (S9–S11 Figs; also see supplementary methods). OneComp eBeads were used to optimise antibody staining panels and apply compensation. For compensation controls, we applied optimal PMT voltages for the positive signal to be detected within 10^4 and 10^5 whereas negative signal set to be below 10^2. Compensation matrices were applied in which there was <40% overlap in any signal combination.

### Fluorescent activated cell sorting

Viable, Anti-F4/80 APC labelled Mϕ or anti-SigLecF+ PE labelled eosinophils, +14d following *Bm*L3 infection, were sorted to >95% purity using a FACS AriaIII Cell Sorter (BD Bioscience, Technology Directorate, UoL).

### Cytospins

Cell suspensions were washed in Hank’s Balanced Salt Solution (HBSS) before being resuspended to a density of 1x10^6^ in HBSS+30% FCS. A volume of 0.1ml was placed in cytospin chambers (Shandon) with poly-l-lysin slides and centrifuged at 450 rpm in a Shandon cytospinner. After air drying, slides were stained with DiffQuick (Shandon) as per manufacturer’s instructions.

### Biochemical & molecular assays

Cellular arginase activities were measured as previously described[55] with the following modifications: 0.25x10^6^ cell suspensions were determined following lysis and protein extraction by enzymatic conversion of arginine to urea, quantified by photometric assay at 570nm (VarioSkan, Bio-Rad). *Arg1* expression levels were determined by RNA extraction of 0.1x10^6^ cell suspensions, reverse transcription and cDNA qPCR transcript analysis using murine TaqMan primers (Applied Biosystems). Data was normalised to *β-act* by the ΔΔCt method.

### *In vivo* treatments

Clodronate liposome suspension (5mg/ml) was diluted 1:5 in PBS and administered 100μl ip 1–3 days prior to infection. αCCR3 was purified from hybridoma supernatant by protein G affinity chromatography (GE Healthcare) and administered at 0.5mg/mouse ip. IL-4c was prepared as previously described [26] and administered at dosages of 1μg rIL-4 ip (unless otherwise stated) at +0, +2 & +4 dpi. CCR3 inhibitor SB328437 (R&D Systems, UK) was administered p.o. at 10 mg/kg *qd* in 1% DMSO PBS between -1-+6dpi.

### *In vitro* cultures

*Bm*L3 were washed in RPMI wash medium containing 1x penicillin, streptomycin and amphotericin B (Life Technologies, UK), before being transferred in batches of 10 *Bm*L3 to 96-well culture plate wells containing RPMI wash + 10% foetal calf serum and 1% normal mouse serum. 1x10^6^ purified eosinophils, Mϕ or eosinophils + Mϕ were added to a total volume of 0.2ml. Cultures were incubated for +7d and motility assessed daily by microscopy.

### Statistical analysis

Significant differences between groups evaluated by Mann-Whitney or Kruskal-Wallis with Dunn’s post-hoc tests (>2 groups). Significance is indicated *P<*0.05* *P<*0.01** *P<*0.001***.

## Supporting information

S1 Fig CCR3 is required to control fecund *B*. *malayi* infection. Total peritoneal microfilariae (mf) enumerated from peritoneal lavage **(A)** or percentage of mice with fecund infections **(B)** in BALB/c WT or CCR3^-/-^ mice, 84 days post-ip infection with 50 *Bm*L3. Data from individual mice with median and interquartile range are plotted. Significant differences between infected groups is assessed by Mann-Whitney (A) or Fisher’s Test (B). Data plotted is pooled from 2 individual experiments and groups of 5–6 mice.
(TIFF)

S2 Fig Macrophage-specific *arg1* transcription levels increase post-infection with *B*. *malayi*. Data plotted is relative expression (median) levels of *arg1* within 0.1x10^6^ FACS purified F4/80^+^ peritoneal Mϕ derived from groups of 3 naïve WT BALB/c mice or +6 days post-ip infection with 50 *Bm*L3. Significant differences between groups is assessed by Mann-Whitney.
(TIFF)

S3 Fig *B*. *malayi* infection induces systemic Th2 responses. Protein levels of IL-4 (A), IL-5 (B) or IL-13 (C) in splenocyte cultures stimulated with soluble *Bm*L3 extract derived from naïve WT BALB/c mice or +6 days post-ip infection with 50 *Bm*L3. Data from individual mice with median levels are plotted. Significant differences between naïve or infected WT groups is assessed by Mann-Whitney. Data is from an individual experiment with groups of 5 mice per group.
(TIFF)

S4 Fig Dose-dependent and -independent effects of exogenous rIL-4 on peritoneal macrophages and eosinophils. Total peritoneal cell **(A)** macrophage **(B)** or eosinophil number **(C)** and peritoneal cell arginase activity **(D)** +4 days following rIL-4c treatment ip on d0 and +2d at indicated doses in BALB/c SCID mice. Data from individual mice with median levels and IQR plotted. Significant differences between IL4c dosed groups is assessed by Kruskal-Wallis with Dunn’s tests. Data is from an individual experiment with groups of 4 mice per group.
(TIFF)

S5 Fig Adaptive Th2 responses remain intact following clodronate liposome treatment. Protein levels of IL-4 (A), IL-5 (B) or IL-13 (C) in splenocyte cultures stimulated with soluble *Bm*L3 extract derived from naïve WT BALB/c mice or WT mice either treated or untreated ip with clodronate liposomes (CL) and subsequent +6 days post-ip infection with 50 *Bm*L3. Data from individual mice with median levels and interquartile range are plotted. Significant differences between naïve or infected WT groups is assessed by Kruskal-Wallis + Dunn’s tests. Data is from an individual experiment with groups of 4–5 mice per group.
(TIFF)

S6 Fig Clodronate liposomes affect proportions of multiple leukocyte populations local and distal to the site of *B*. *malayi* infection. Flow cytometric determination of peritoneal neutrophil or B cell numbers in BALB/c WT mice +6dpi following inoculation ip with 50*Bm*L3 with or without prior ip CL treatment **(A-D).** Proportions of circulating monocytes in naïve BALB/c WT mice or in BALB/c WT mice +6 days following ip CL treatment **(E-F)**. Data from individual mice with median levels and interquartile range are plotted. Significant differences between naïve or infected WT groups is assessed by Mann-Whitney tests. Data is from an individual experiment with groups of 5 mice per group.
(TIFF)

S7 Fig Establishment of adoptively transferred *Bm*L3AAMϕ in the peritoneum of clodronate treated WT or SCID mice. Numbers of peritoneal Mϕ at indicated time points in BALB/c WT mice **(A)** or SCID mice **(B)** +/- pre-treatment with clodronate liposomes (CL) and subsequent +/- adoptive transfer of 0.75x10^6^ *Bm*L3AAMϕ coincident with inoculation with 50 *Bm*L3. Cellular arginase activity in BALB/c SCID mice +/- adoptive transfer of 0.75x10^6^ WT *Bm*L3AAMϕ +14 days post infection with 50 *Bm*L3. Data from individual mice with median levels are plotted. Significant differences between naïve or infected WT groups is assessed by Mann-Whitney tests. Data is from an individual experiment or pooled from two experiments, with groups of 4–6 mice per group.
(TIFF)

S8 Fig CCL11 and CCL24 chemokine transcript analysis in peritoneal cells post infection with *B*. *malayi*. Relative transcript levels of *ccl11* **(A)** or *ccl24* **(B)** in BALB/c WT or IL-4Rα^-/-^ mice + 6 days post-infection with 50 *Bm*L3. Data plotted is relative expression (median +IQR) levels of specific transcripts within 0.1x10^6^ peritoneal cells derived from groups of 5 mice. Significant differences between groups is assessed by Mann-Whitney tests.
(TIFF)

S9 Fig Schematic of peritoneal eosinophil and macrophage flow cytometric gating strategy. (TIFF)

S10 Fig Schematic of peritoneal neutrophil and B-cell flow cytometric gating strategy. (TIFF)

S11 Fig Schematic of blood monocyte flow cytometric gating strategy. (TIFF)

S12 Fig Graphical summary. (TIFF)
