# Supplementary figures and images for: Interleukin-4 activated macrophages mediate immunity to filarial helminth infection by sustaining CCR3-dependent eosinophilia
Source: PLoS Pathog. 2018 Mar 16;14(3):e1006949. doi: 10.1371/journal.ppat.1006949 (PMC5874077; doi:10.1371/journal.ppat.1006949)

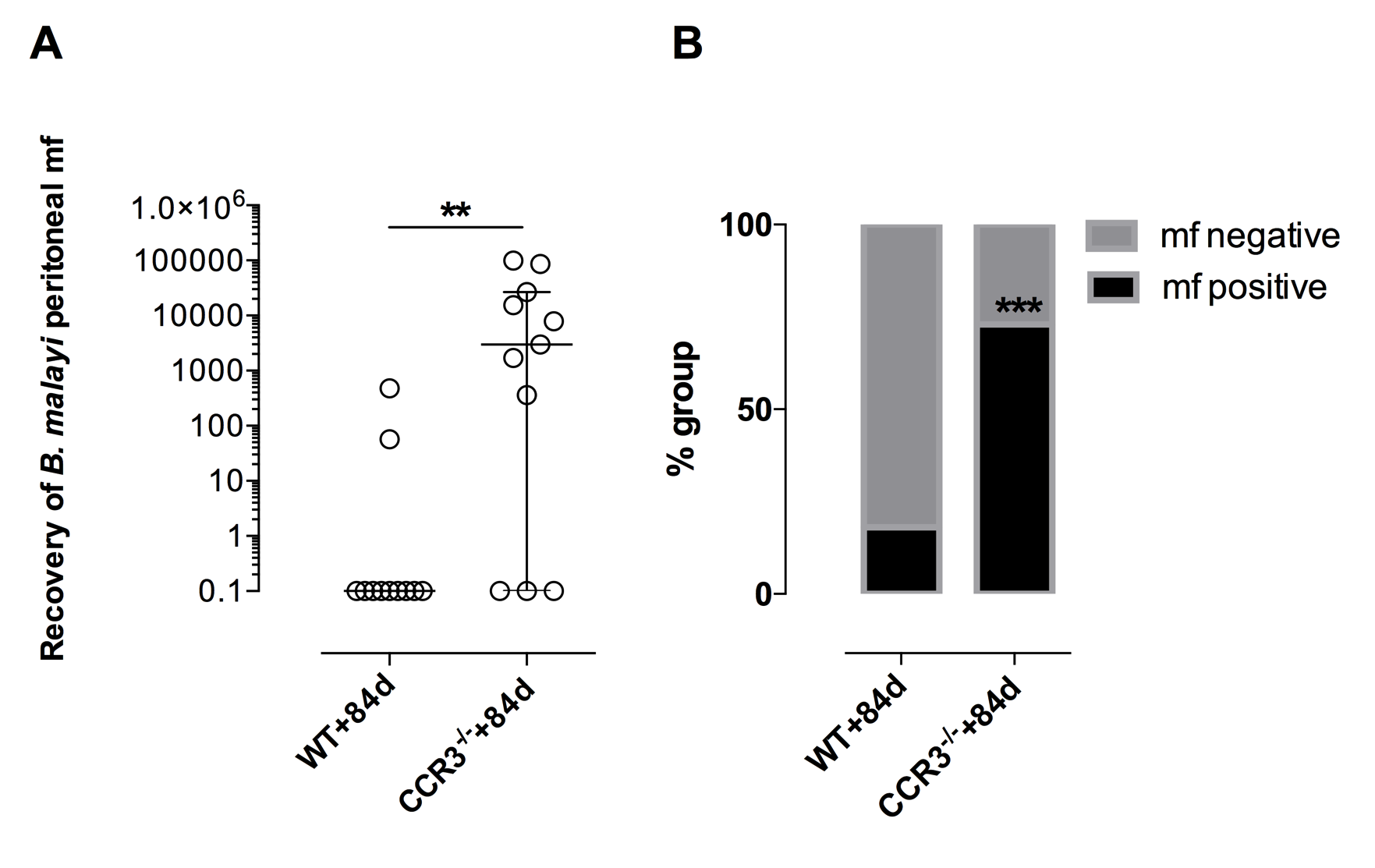

Supplement: S1 Fig — Total peritoneal microfilariae (mf) enumerated from peritoneal lavage (A) or percentage of mice with fecund infections (B) in BALB/c WT or CCR3-/- mice, 84 days post-ip infection with 50 BmL3. Data from individual mice with median and interquartile range are plotted. Significant differences between infected groups is assessed by Mann-Whitney (A) or Fisher’s Test (B). Data plotted is pooled from 2 individual experiments and groups of 5–6 mice. (TIFF) [file ppat.1006949.s001.tiff]

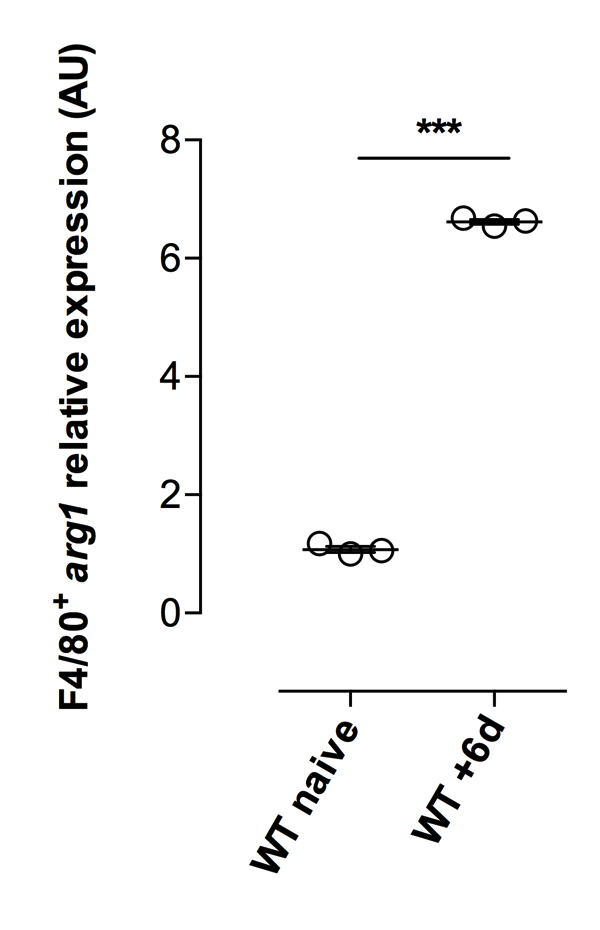

Supplement: S2 Fig — Data plotted is relative expression (median) levels of arg1 within 0.1x10^6^ FACS purified F4/80+ peritoneal Mϕ derived from groups of 3 naïve WT BALB/c mice or +6 days post-ip infection with 50 BmL3. Significant differences between groups is assessed by Mann-Whitney. (TIFF) [file ppat.1006949.s002.tiff]

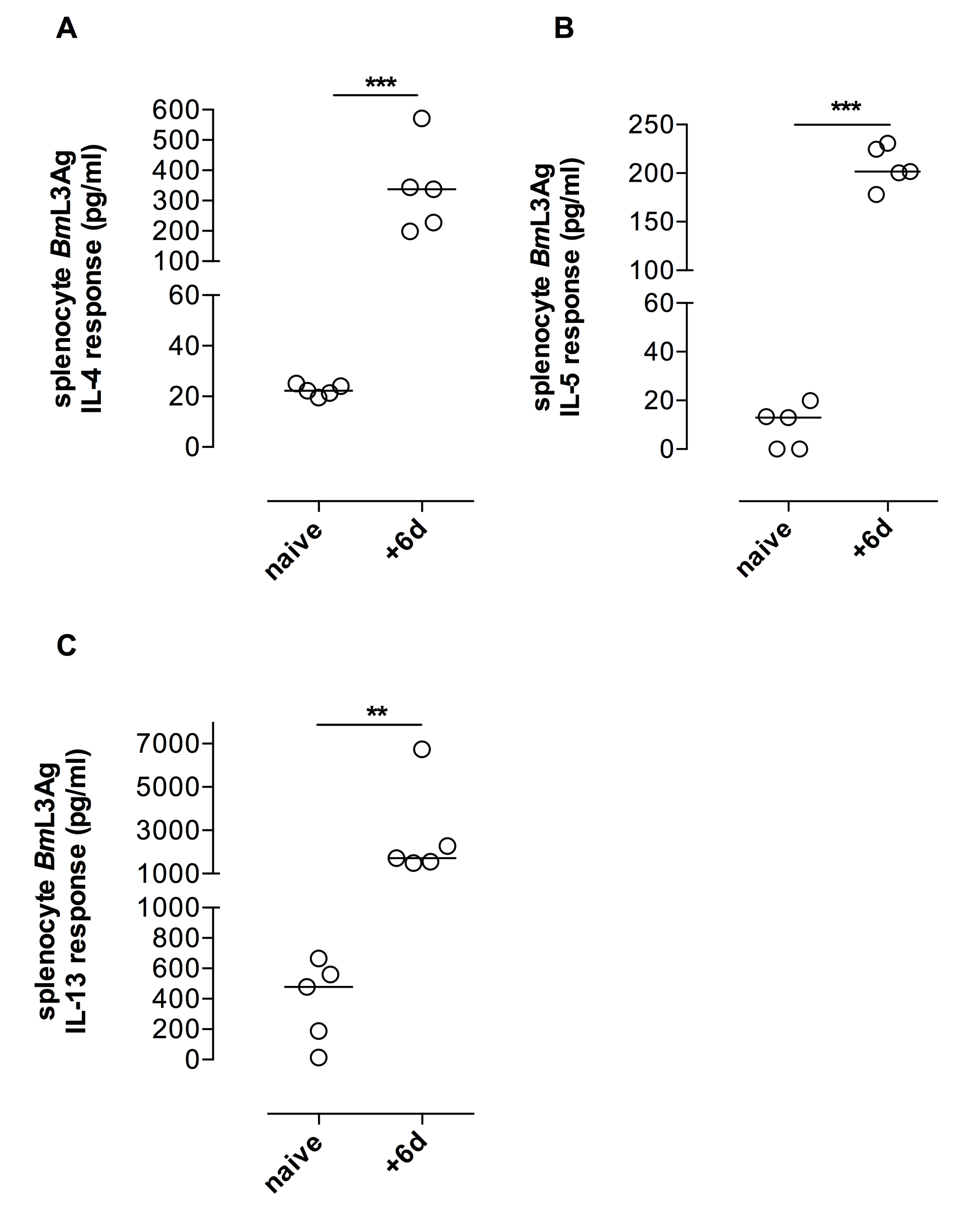

Supplement: S3 Fig — Protein levels of IL-4 (A), IL-5 (B) or IL-13 (C) in splenocyte cultures stimulated with soluble BmL3 extract derived from naïve WT BALB/c mice or +6 days post-ip infection with 50 BmL3. Data from individual mice with median levels are plotted. Significant differences between naïve or infected WT groups is assessed by Mann-Whitney. Data is from an individual experiment with groups of 5 mice per group. (TIFF) [file ppat.1006949.s003.tiff]

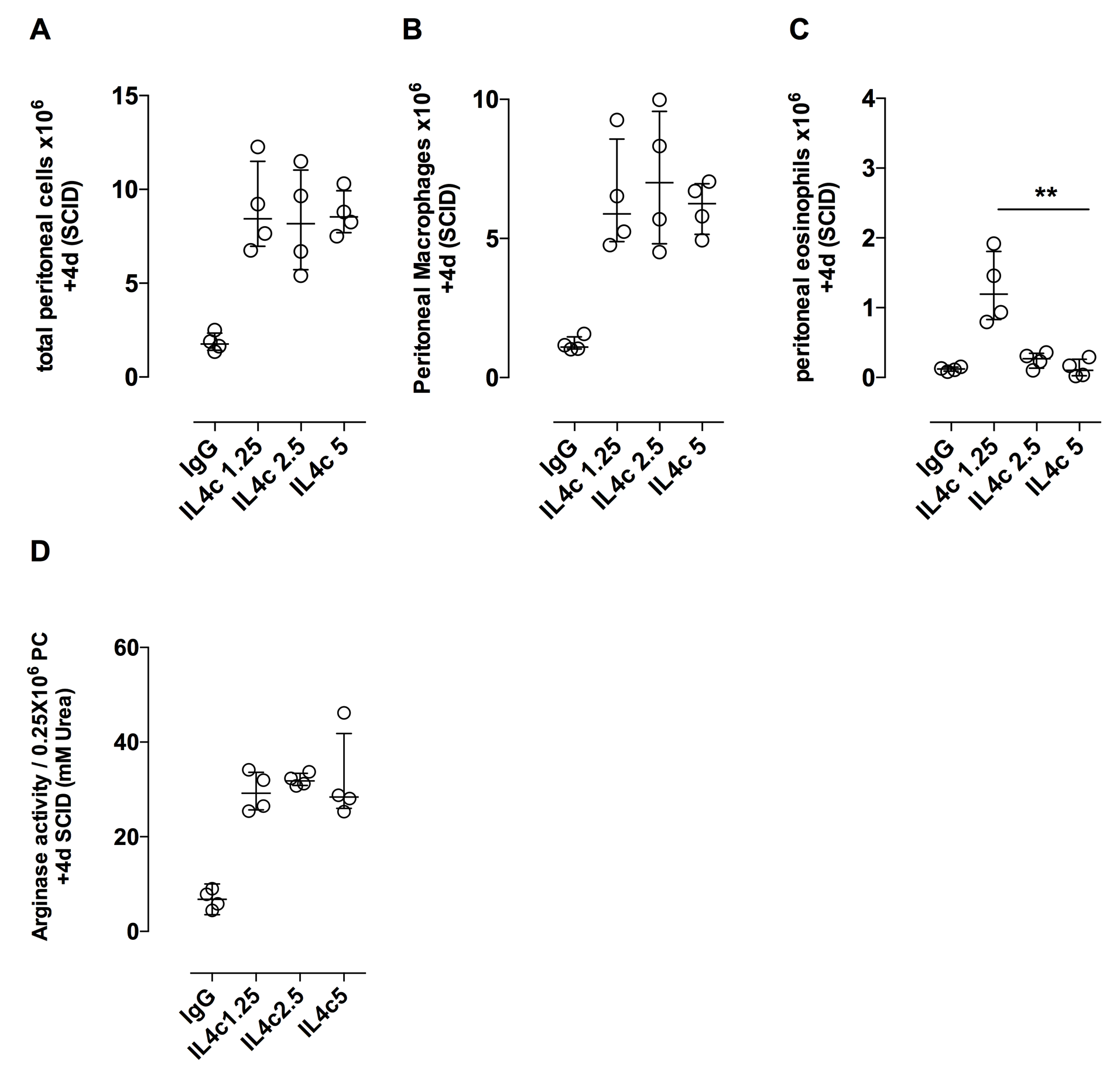

Supplement: S4 Fig — Total peritoneal cell (A) macrophage (B) or eosinophil number (C) and peritoneal cell arginase activity (D) +4 days following rIL-4c treatment ip on d0 and +2d at indicated doses in BALB/c SCID mice. Data from individual mice with median levels and IQR plotted. Significant differences between IL4c dosed groups is assessed by Kruskal-Wallis with Dunn’s tests. Data is from an individual experiment with groups of 4 mice per group. (TIFF) [file ppat.1006949.s004.tiff]

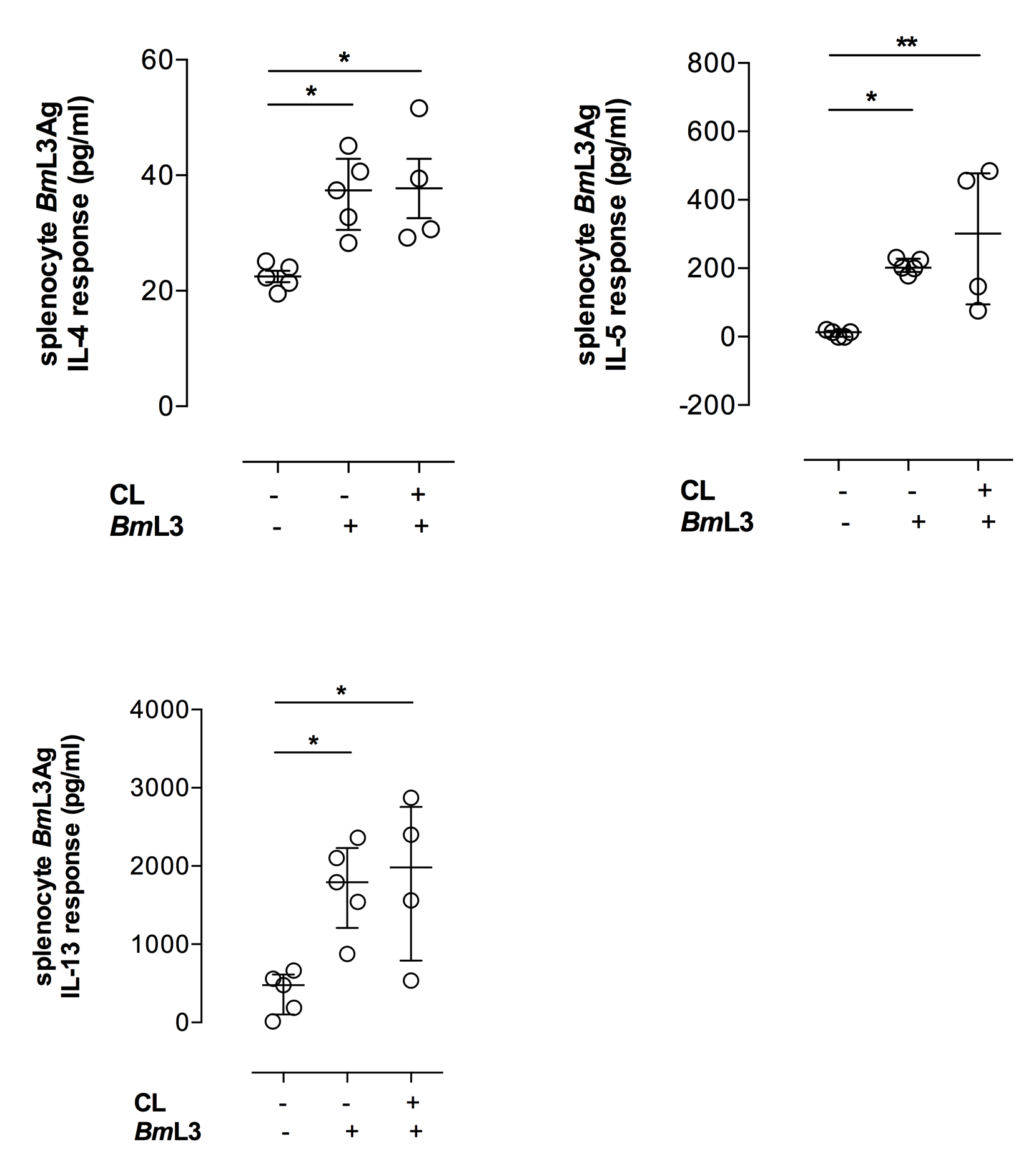

Supplement: S5 Fig — Protein levels of IL-4 (A), IL-5 (B) or IL-13 (C) in splenocyte cultures stimulated with soluble BmL3 extract derived from naïve WT BALB/c mice or WT mice either treated or untreated ip with clodronate liposomes (CL) and subsequent +6 days post-ip infection with 50 BmL3. Data from individual mice with median levels and interquartile range are plotted. Significant differences between naïve or infected WT groups is assessed by Kruskal-Wallis + Dunn’s tests. Data is from an individual experiment with groups of 4–5 mice per group. (TIFF) [file ppat.1006949.s005.tiff]

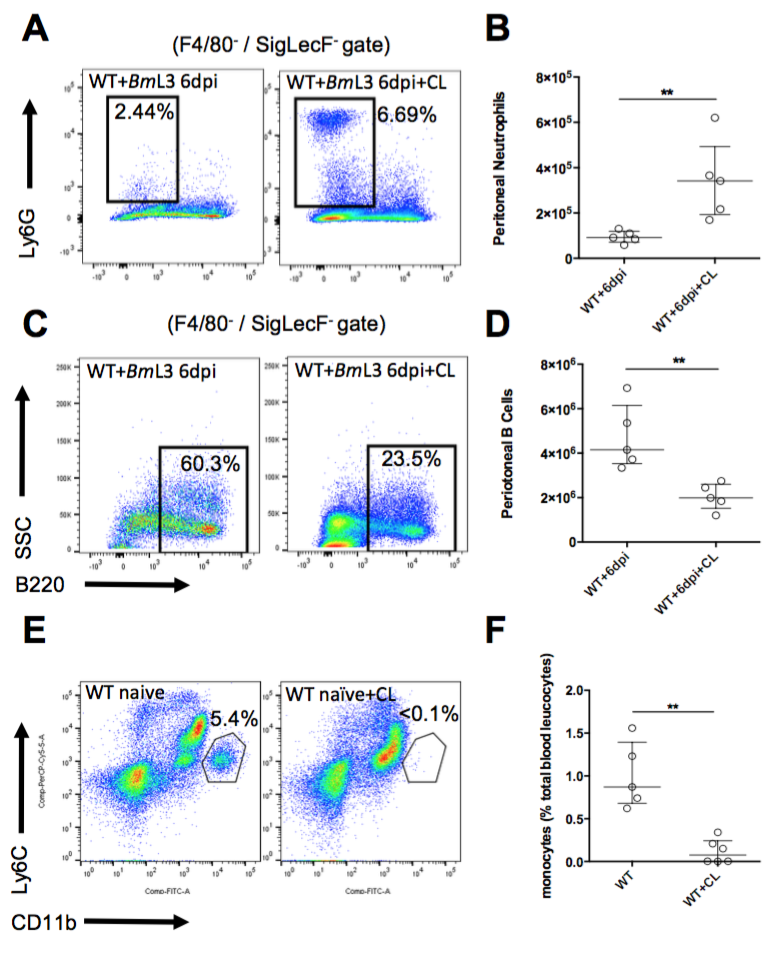

Supplement: S6 Fig — Flow cytometric determination of peritoneal neutrophil or B cell numbers in BALB/c WT mice +6dpi following inoculation ip with 50BmL3 with or without prior ip CL treatment (A-D). Proportions of circulating monocytes in naïve BALB/c WT mice or in BALB/c WT mice +6 days following ip CL treatment (E-F). Data from individual mice with median levels and interquartile range are plotted. Significant differences between naïve or infected WT groups is assessed by Mann-Whitney tests. Data is from an individual experiment with groups of 5 mice per group. (TIFF) [file ppat.1006949.s006.tiff]

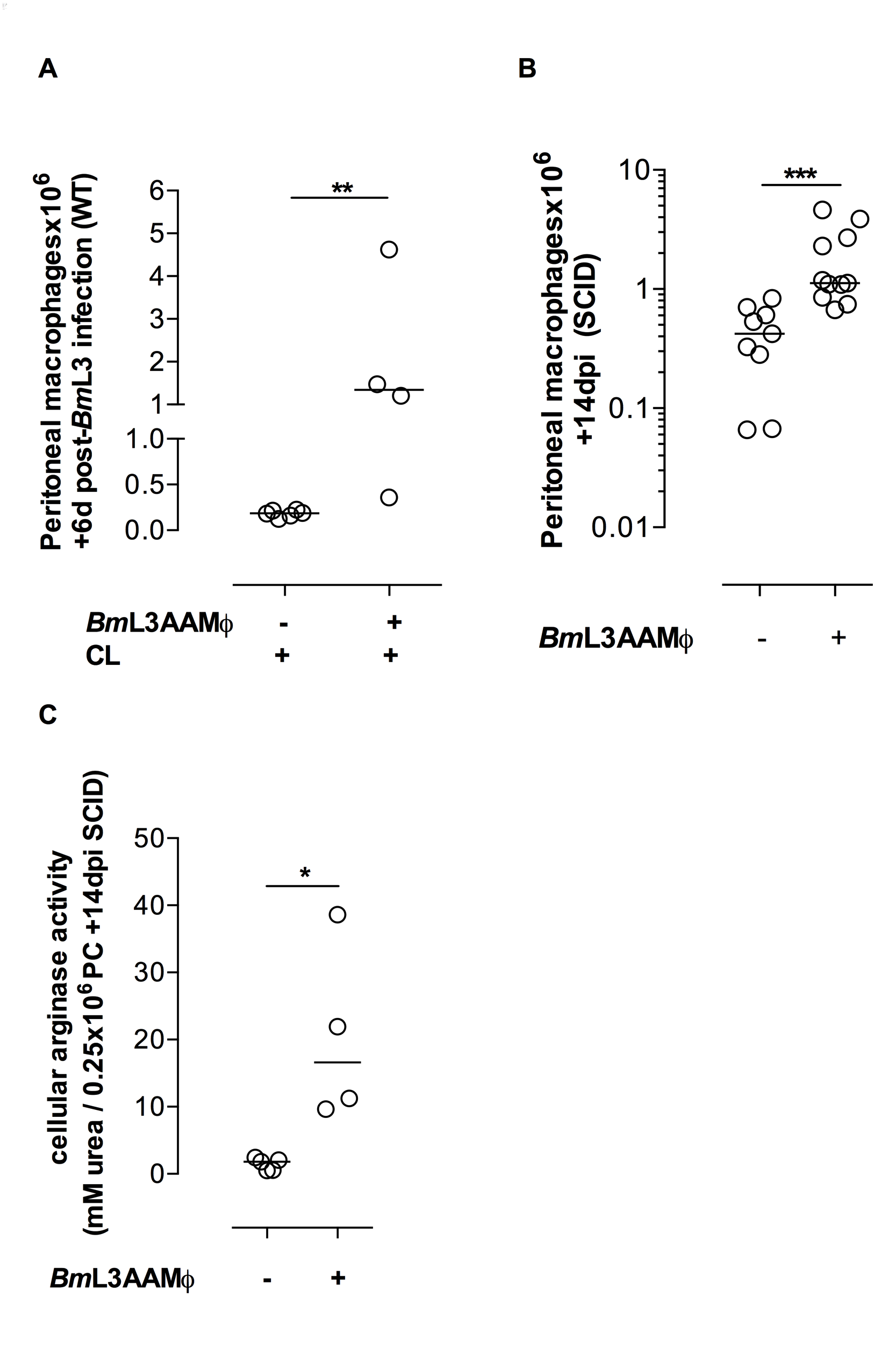

Supplement: S7 Fig — Numbers of peritoneal Mϕ at indicated time points in BALB/c WT mice (A) or SCID mice (B) +/- pre-treatment with clodronate liposomes (CL) and subsequent +/- adoptive transfer of 0.75x10^6^ BmL3AAMϕ coincident with inoculation with 50 BmL3. Cellular arginase activity in BALB/c SCID mice +/- adoptive transfer of 0.75x10^6^ WT BmL3AAMϕ +14 days post infection with 50 BmL3. Data from individual mice with median levels are plotted. Significant differences between naïve or infected WT groups is assessed by Mann-Whitney tests. Data is from an individual experiment or pooled from two experiments, with groups of 4–6 mice per group. (TIFF) [file ppat.1006949.s007.tiff]

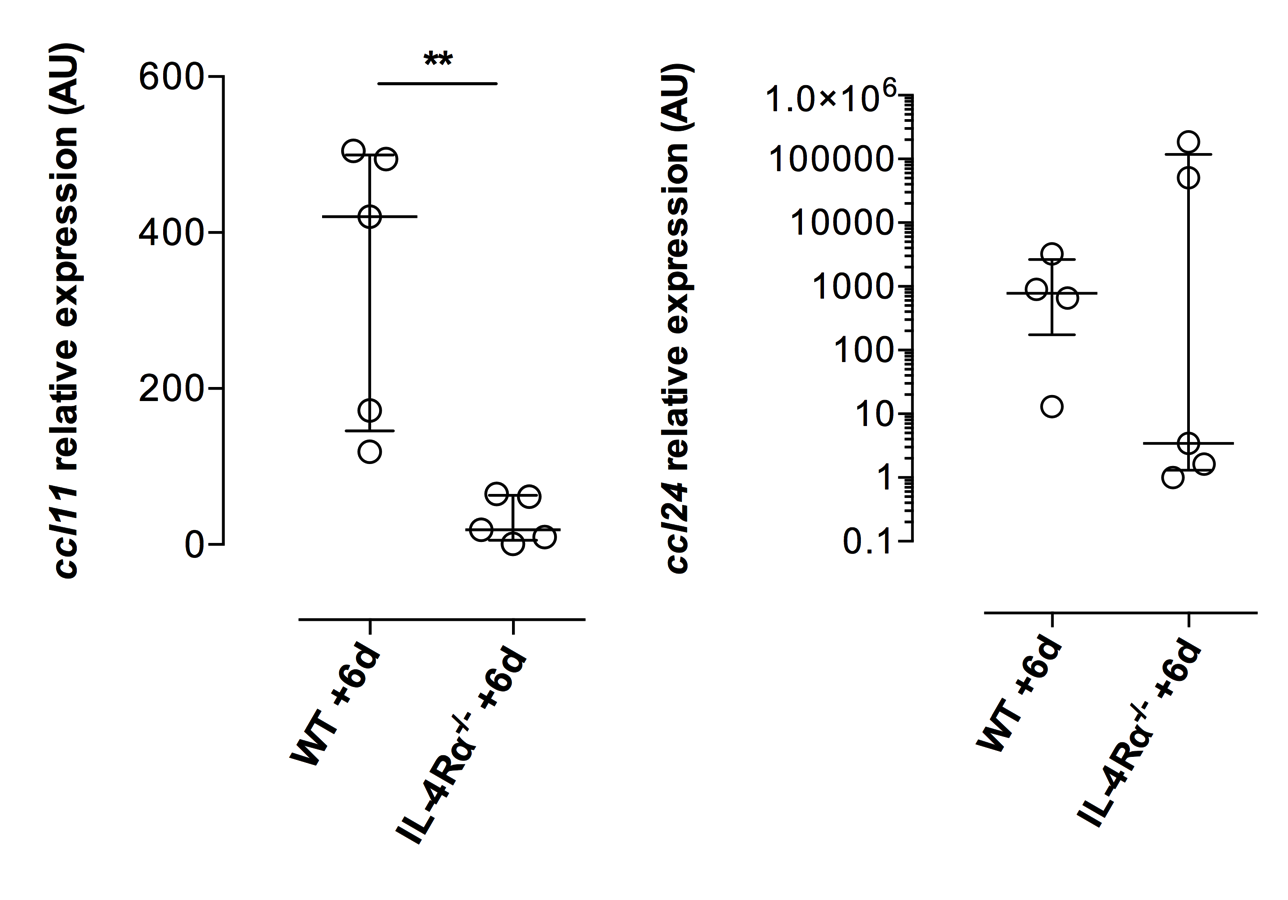

Supplement: S8 Fig — Relative transcript levels of ccl11 (A) or ccl24 (B) in BALB/c WT or IL-4Rα-/- mice + 6 days post-infection with 50 BmL3. Data plotted is relative expression (median +IQR) levels of specific transcripts within 0.1x10^6^ peritoneal cells derived from groups of 5 mice. Significant differences between groups is assessed by Mann-Whitney tests. (TIFF) [file ppat.1006949.s008.tiff]

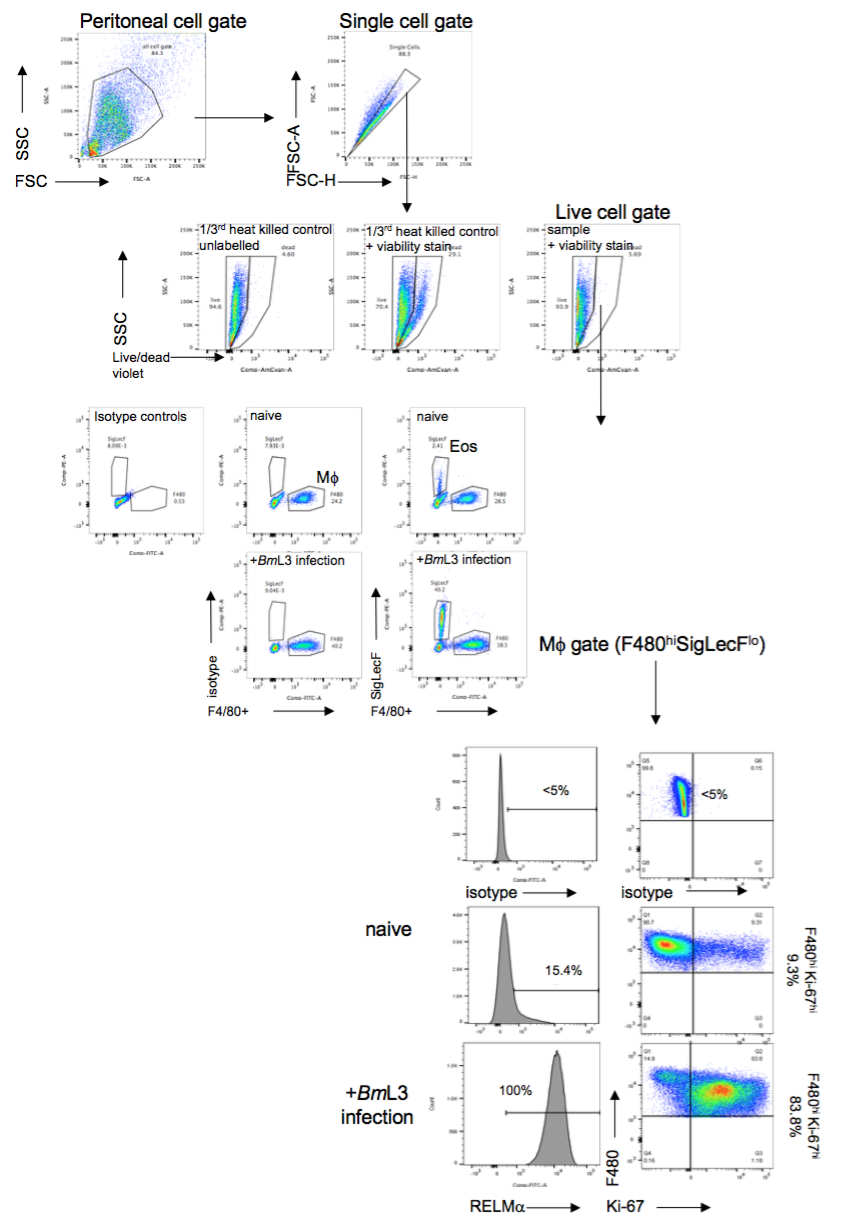

Supplement: S9 Fig — (TIFF) [file ppat.1006949.s009.tiff]

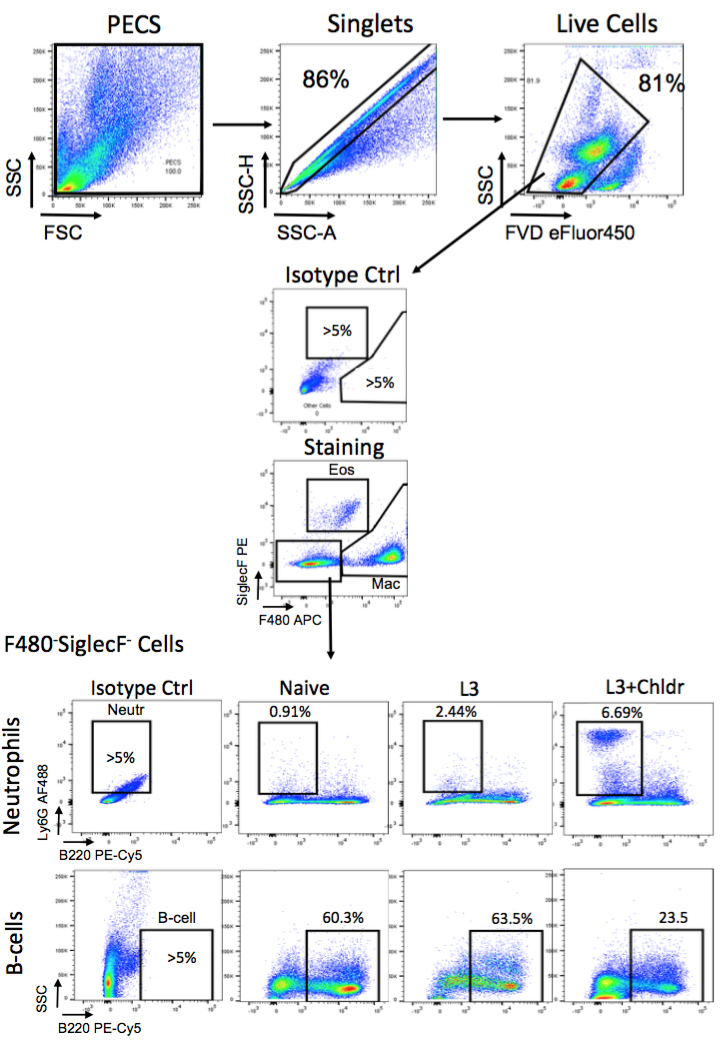

Supplement: S10 Fig — (TIFF) [file ppat.1006949.s010.tiff]

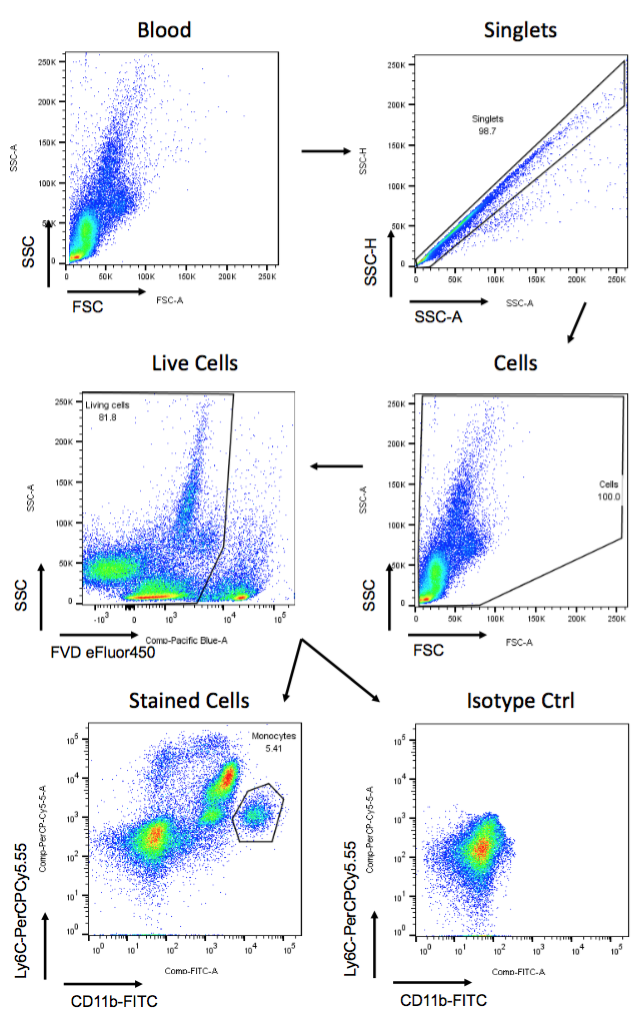

Supplement: S11 Fig — (TIFF) [file ppat.1006949.s011.tiff]

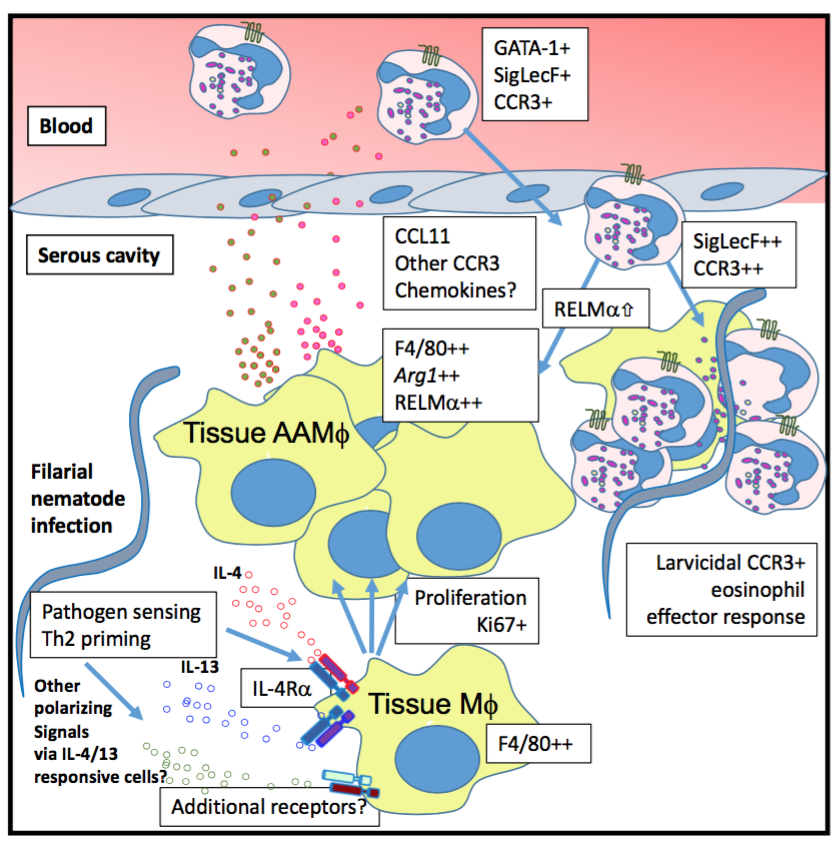

Supplement: S12 Fig — (TIFF) [file ppat.1006949.s012.tiff]
